# Implications to the electricity system of Paraguay of different demand scenarios and export prices to Brazil

**DOI:** 10.1007/s12667-020-00420-w

**Published:** 2021-01-11

**Authors:** Ioannis Pappis, Carlos Centurion, Eunice Pereira Ramos, Mark Howells, Silvia Ulloa, Eduardo Ortigoza, Pedro E. Gardel-Sotomayor, Thomas Alfstad

**Affiliations:** 1 Department of Energy Technology, Division of Energy Systems, KTH Royal Institute of Technology, Brinellvagen 68, 10044 Stockholm, Sweden; 2 Jefe del Departamento Económico Financiero, Dirección de Formulación Evaluación de Proyectos, Dirección General de Proyectos de Participación Público Privada, Secretaria Tecnica de Planificatión del Desarrollo Económico y Social, Asunción, Paraguay; 3 Department of Geography, Loughborough University, Loughborough, UK; 4 Centre for Environmental Policy, Imperial College London, London, UK; 5 Stockholm Environment Institute, Somerville, USA; 6 Polytechnic School, National University of Asunción, San Lorenzo, Paraguay; 7 Catholic University “Nuestra Señora de La Asunción” Alto Parana Campus, Asunción, Paraguay; 8 United Nations Department of Economic and Social Affairs, New York, NY USA

**Keywords:** Energy planning, Demand scenarios, Cost-optimization, OSeMOSYS, Project finance, Investment outlook

## Abstract

Paraguay's power system is based entirely on hydropower. It serves as the largest net electricity exporter in Latin America. Nonetheless, the country´s electricity consumption per capita is one of the lowest in the world and the transmission and distribution network has one of the highest losses in Latin America. This paper presents an electricity expansion investment outlook (2018–2040) for Paraguay using OSeMOSYS, analyzing three electricity demand scenarios under different electricity export prices to Brazil. The study identifies the least-cost power generation mix, future investments and the financial requirements to meet the needs of different demand scenarios. We find that Paraguay will need to invest in hydropower plants, by mainly expanding the capacity of Yacyreta to cover its electricity needs and sustain national electricity exports levels. In the High demand scenario, where the electricity demand could approximately double by 2040, the country's overall electricity exports decrease by 50% compared to the Reference scenario. Based on the different scenarios examined, the government spends approximately 18.3–31.2 billion USD on power plant investments for the period 2018–2040 to cover future electricity demand. The findings could be useful in supporting decision-making concerning socio-economic development pathways in the country.

**Supplementary Information:**

The online version contains supplementary material available at 10.1007/s12667-020-00420-w.

## Introduction

Access to modern energy services is essential for economic growth and human development [1,2]. The importance of energy for Paraguay is reflected in the government's ambitions to meet core goals in their national energy plans such as energy security, energy equity and environmental sustainability [3,4].

This study focuses on pathways for the development of the national electricity system of Paraguay. Concerning electricity production, the country has certain unique features. It is the country with the highest percentage of renewable energy per capita in the world, the largest net electricity exporter in South America (85% of all electricity generated is exported) and one of the largest net electricity exporters in the world [5,6]. Its domestic primary energy supply is based on hydropower (67%) and biomass (33%) [5]. Electricity production is almost entirely (99.7%) from hydropower, primarily from two large hydro projects, on Yacyreta (3.2 GW) and Itaipu (14 GW) dams [5]. These power plants are co-owned (50% shared installed capacity) between Argentina and Brazil, respectively. Despite this, 75% of the total electricity produced (55,282 GWh, [5]) in Paraguay from these plants is exported to the same two countries. The electricity imports from Itaipu constitute approximately one-fifth of Brazil’s final electricity consumption [7]. The two hydropower plants have a significant contribution to the country's economy, owing to their size as well as the scale of electricity trade between the nations.

The paradox is that Paraguay has one of the lowest electricity consumption per capita values in South America, almost 1563 kWh in 2014 (2nd lowest after Bolivia). The energy service satisfaction level is very low and one-third of the population lives in extreme poverty [8]. The very low electricity tariffs and the high losses in the transmission (4%) and distribution (17%) network of the country make it difficult to increase access due to the poor financial returns, exacerbated by the inefficiency of the transmission and distribution system. Nevertheless, the national electric power distributor ANDE aims to improve the efficiency of the local grid for the period 2016–2025 [7]. Another challenge is the uncertainty of the electricity export prices in the coming years and the domestic revenues generated by those as the power purchase agreement for electricity exports for one of the two projects is due for renegotiation in 2023. Explicitly, Annex C of the Itaipu treaty specifies the electricity export price (45.2 USD/MWh) between Paraguay and Brazil. Brazil pays to Itaipu Binacional, the cost of producing the electricity and a compensation rate of about 9 USD/MWh to Paraguay for using the part of the Paraguayan share of the electricity produced [9],^10^. The electricity generated by Itaipu belongs equally to Paraguay and Brazil. Since Paraguay cannot use all the energy that is entitled to, by treaty, it must cede what it does not use to Brazil, which pays Paraguay a fixed cession rate in addition to the generation cost. The Annex C was signed in April 1973 and will be renegotiated in 2023. The result of the negotiations will affect the country´s future economic and social development. One of this paper's objectives is to model aspects of this electricity export price uncertainty and assess its impact on all the other energy decision-making needed to meet the domestic electricity demands (under different scenarios) until 2040. The three demand scenarios based on different electricity growth (Reference, Medium, High) for the analyses have been selected given the dynamic nature of a developing economy like Paraguay's.

Paraguay faces particular other challenges related to its overall energy consumption. The country doesn´t have any domestic fossil fuel reserves and relies entirely on imports for the different sectors (e.g., the transport sector) [7]. Also, for residential energy use, the consumption of traditional biomass is significantly high, a contributing cause to deforestation [5].

Long-term energy modeling and tools can explore dynamics and associated risks in undertaking large-long term investments in the power sector (e.g., use of energy reserves, affordability, energy security, finances, capacity investments) to meet a nation´s domestic growing demand in the future. There are various modeling tools for energy planning [11–16]. Two wide-spread modeling approaches are also categorized in top-down and bottom-up, and there are existing studies that integrate those two modeling approaches [17–24]. The top-down models focus on the broader economy and the macro-economic relationship of the system´s components with the energy sector and incorporate feedback effects between different markets, considering changes in prices and incomes. The bottom-up models focus primarily on the energy system (e.g., energy supply processes, conversion technologies, end-use demand patterns) and are technology-specific, framing around mathematical programming problems. They do not account for price distortion or economy-wide interactions and income effects [25],^26^.

To address the challenges mentioned above, this study focuses primarily on Paraguay’s electricity supply system. It provides insights for strategic planning by producing cost-optimal development pathways for the electricity system. Under different electricity demand scenarios, it provides a cost-optimal power generation mix to meet plausible future local/regional electricity demands. Identifying a cost-optimal generation mix, may help Paraguay address the question of how to better use its hydropower electricity for the country´s socio-economic development. Analyzing the implications of the demand risk for the government of Paraguay and Itaipu dam under the different demand and electricity export prices scenarios is essential for long term energy planning. As the Itaipu debt is expected to be paid by 2023, the future development of the electricity export price of Itaipu will affect the balance of electricity exports to other countries and the total revenues for the government would be beneficial to be examined. The study uses an open-source cost-optimization tool for medium to long-term energy planning (OSeMOSYS).

The paper is organized as follows. In Sects. 1.1 and 1.2, there is a brief introduction to the country´s economic context and its energy-related issues and policies and later a literature review on decision analysis and energy planning for reforming national policies. The methodology of the model development and the different demand scenarios analyzed are presented in Sect. 2. In Sect. 3, the results are investigated and discussed, and in Sect. 4, the conclusions and policy implications of this study are presented.

### Country background and energy issues

Paraguay is a land-locked country located in South America. It shares a border to the east with Brazil, to the south-west with Argentina and to the north with Bolivia.

The economy of Paraguay has experienced a steady growth with an average GDP growth rate of 5% for the period 2010–2017 [8]. Nevertheless, it is still the second poorest nation in South America after Bolivia [8]. The service sector represents most of the country´s economy, 48% of the GDP in 2018. Table 1 presents the profile of the country.

**Table 1:** Country profile

Capital	Asunción
Region	South America
Surface area (sq. km)	406,752
Population (mil.)	6.956 (2018)
Population—capital (mil.)	3.222 (2018)
Population density (people per sq.km)	17.5 (2018)
Urban population (% of the total)	61.6%
Population—Annual growth rate	1.3%
GDP per capita (current US$)	5,822 (2018)
GDP growth (annual %)	3.7% (2018)
Electrification rate (% of population)	99.3% (2017)

The total primary energy supply of Paraguay was 295 PJ in 2017, with hydro constituting around 20%, biofuels and waste 44%, oil products 36% and coal less than 1%. In that year, electricity exports accounted for approximately 53% of the country´s total energy supply, presenting an average annual increase of 1% the period 2010–2017, while the charcoal exports around 1%. During that period, the domestic electricity supply increased with an average growth rate of 6% while the share of the electricity exports out of the domestic electricity supply decreased with an average rate of 5%. Paraguay´s imports were 105 PJ in 2017, of which the oil product’s share was 99.96% and charcoal´s share 0.04%. The total final consumption was 266 PJ in 2017 with the transport sector to account for most of it (40%), followed by the residential (28%), industrial (25%) and commercial and other (7%) sectors. In the transport sector, the country has started using biofuels with E25 as a blending mix for bio-ethanol and 1% for biodiesel to decrease fossil fuel emissions by 20% by 2030 [5,27,28].

The total installed capacity of the country was 8844 MW in 2017, with hydro constituting the majority (99.7%). The electricity system of Paraguay is mainly powered by two binational (Itaipu, Yacyreta) and one national (Rio Acaray) hydropower plant. The Parana River, located in the Southeastern area of the country, is responsible for most of this hydroelectric generation potential. The Itaipu Binacional hydroelectric dam is the 2nd largest operational hydroelectric energy producer in the world and a cornerstone of the country´s energy system [29]. The guaranteed electricity production in Itaipu is 75 million MWh, but the average production is around 90 million MWh. According to the Itaipu Annual report, the production exceeds the nominal capacity of generating units mainly because of its operation and maintenance care. In 2019, one of the driest years since the beginning of the operation, Itaipu produced a total of 79,444,510 MWh. In 2016, the production reached a total of 103,098,366 MWh, a new world record in the annual generation. Its previous record was reached in 2013, with 98,630,035 MWh [30].

The participation of Itaipu electricity in the Paraguayan market has been increased from 73% in 2012 to almost 90% in 2019. This gradual increase emphasizes the importance of Itaipu´s electricity supply to the Paraguayan electricity market. However, Itaipu´s supply in the Brazilian market has been decreasing throughout the years due to the rise of the Brazilian demand and the increase in the use of Paraguayan electricity [30].

The large and continuous flow of the Parana River, and the good maintenance and operation records of the enterprise, makes it possible for Itaipu to supply over 10% of the Brazilian electricity demand and 80% of the Paraguayan demand all year round. The decrease in generation during the winter months corresponds to the decline in demand [30].

The hydropower production is reflected in the very low electricity tariffs in the country (0.054–0.076 USD/kWh) [31]. The total electricity demand was 11,560 GWh in 2017. The residential sector was the primary electricity consumer (43%), followed by commercial and public services (37%), and industry (20%) one [8].

Despite Paraguay having an available hydroelectric surplus and an estimated hydropower potential of 56 GW the western region of Paraguay often has difficulty in accessing electricity due to the geographical location of the electricity generating plants in this part of the country [32]. The population in this region must burn fossil fuels, or look for other ways, to satisfy the demand for electricity despite the availability of solar and wind energy potential [33, 34]. The country could also invest in renewable energy sources other than hydropower. However, several times throughout the year, solar and wind energy is limited, so the population in this region must proceed to the burning of petroleum derivatives, or other sources, which leads to the emission of CO_2_. This issue, along with indoor air pollution, often prevents improving the quality of life of society in that area of the country.

Furthermore, the solar and wind energy potential could be used as alternatives technologies for electricity exports to other countries [6,17,32]. However, the main challenge is sourcing foreign investment, given the low financial returns (low regulated electricity tariffs).

### Large hydropower projects as the backbone of energy systems

Investments in large hydropower projects are associated with socio-economic impacts and environmental benefits and risks [35–37]. Investing in large hydropower projects for regional development through cross-border electricity interconnection projects can offer opportunities as well as risks. A risk associated with undertaking large long-term investments in the country´s energy policy plan is the implication of sudden shifts to other potential technologies to meet the country´s domestic growing demand in the future.

Yet, much is to be gained from the exploitation of hydropower. In the case of Paraguay, countries relying on large hydropower projects face particular vulnerabilities concerning energy security, socio-economic development and geopolitical relationships (Itaipu Treaty, [9]). In the case of Brazil, the country generates around 80% of its electricity from hydropower (83 GW) [5] and, in its energy expansion plan for 2011–2020, planned to increase this capacity by around 20 GW, investing in 30 additional large dams in the Legal Amazon region. However, several of those plants will be financed and built by Brazil in Peru, Bolivia, Ecuador and Guyana without taking into consideration environmental (water availability in river basins) and social impact in the Amazonia region [38].

In Paraguay, the main source of electricity has been and will continue to be that of hydropower. Besides, there is potential that has not been exploited yet with Argentina [39]. Indeed, the hydroelectric energy generated by Itaipu, the part corresponding to Paraguay, could be used to promote national development but from a different approach than the traditional one [40]. The energy transition is being promoted to replace oil derivatives with renewable resources.

Quantitative studies using optimization modeling have been used to investigate the issues above (socio-economic viability, environmental concerns) for energy development and provide insights for long-term planning, electricity trades and policy implications [14]. One of those is the South America Model Base (SAMBA) developed in OSeMOSYS [41]. The analysis examined the transformation of the overall generation mix in the continent under different electricity trade scenarios taking into consideration strategic large hydropower plants. Also, the study highlighted the cross-border potential electricity trade that Bolivia could have with neighboring countries by investing in large hydropower power plants [42]. The analysis above does not include the annual revenues from the respective countries' electricity exports, which could be used by each government to finance the upcoming power plant projects. Also, the SAMBA model could be soft-linked with a project finance input–output model to estimate the potential annual payments for each government from electricity exports of a specific hydroelectric power plant. Both of those concepts are analyzed in our study. A continental electricity trading scheme such as the SAMBA one could complement our research to identify electricity trades with other countries except for the ones to Argentina and Brazil. Moreover, in our study, the SAMBA study's techno-economic assumptions for Paraguay and the list of power plants considered are updated with the latest ones.

Another study investigated the local socio-economic impacts (GDP, public revenues) of large hydropower plant development in a developing country (Brazil as a case study) using an econometric approach [43]. Based on this research, the country will boost its economy in the short-term during the construction of the hydropower plants. However, in the long-term, the effect will be low, with little to no improvement in socio-economic conditions. Besides, investing in small hydropower plants was reported to have a more favorable local impact than larger plants, especially for agricultural GDP. This paper focuses only on the local effects of site construction and does not evaluate the overall effects of the electricity transmitted to other parts of the Brazilian economy [43]. A similar study could be conducted for Paraguay, which is missing from the current literature and linked with our research outcomes, specifically the future identified hydropower plants and the transmitted electricity.

Another study applied a Multi-Criteria Decision Analysis based on the Analytic Hierarchy Process considering four policy options for Paraguay to investigate the most promising ones from economic, technical, political, social and environmental points of view [44]. An outcome of this study was that the most promising policy options to affect the society of Paraguay positively are the establishment of small industries and allow for high electro-intensive industry penetration levels. Also, since the power system of the country is mainly based on the two-large binational hydropower plants (Yacyreta, Itaipu), it makes it difficult for the state to diversify its power generation mix at the future. However, this study only focused on which policy option could be the most favorable one to exploit the excess electricity to Brazil. As in our research, a cost-optimization modeling approach was used to identify any potential cost-optimal future implementation of new power plants and how, through different electricity export regimes, the generation from Itaipu could shift to either cover domestic electricity demand or increase electricity exports. An integrated study of those two could better understand the most promising policy option considering the specific annual levels of surplus electricity among a framework of scenarios with changes in electricity demand and export prices.

The study on learning lessons from Paraguay’s productive hydropower system proposed that the country should invest in high consuming energy industries instead of electricity exports [45]. In our analysis, we identify by using a cost-optimization energy systems modelling framework the trade-off between electricity exports and different electricity demand projections and indicate the annual revenues which could be used for different purposes based on governments policy plans (e.g. invest in high consuming energy industries). In that way, in the high electricity demand scenario (HED) that considers the penetration of high electro-intensive industries in the country the government can identify the financial and technical-power generation capacity implications on its future energy transition. Annex C of the Itaipu treaty will be revised in 2023. A study investigated different scenarios related to electricity export prices between Paraguay and Brazil and how those will affect the country´s social and economic conditions [46]. However, in that study, a bottom-up simulation tool for Long-range Energy Alternatives Planning system (LEAP) was used, a version of the tool that is not suitable for financial planning and to identify least-cost policy solutions since is based on demand-driven [47]. The novelty in our analysis is that we applied a cost-optimization tool (OSeMOSYS) [48,49] for long-term energy planning to identify the cost-optimal power generation mix and considered different scenarios related to electricity export prices between Paraguay and Brazil and different electricity demand scenarios to provide a broader understanding of the energy transition.

Among others, a study analyzed the economic, social and environmental impact, which involves reducing the export of hydroelectric energy and influences the need for industrialization in the country, identifying which are the most complex productive sectors according to the theory product space [50]. In this study, the social criterion taken into account corresponds to the number of jobs that could be generated with the implementation of the selected industry. However, for the environmental criterion, the greenhouse gas emission rate (CO2) was considered and for the economic criterion, the Revealed Comparative Advantage (VCR) index was considered. This is not considered in our analysis. Nevertheless, our study can complement the previous one by identifying the future installation of power plants and the associated job creation [51] and the drivers of the electricity demand (e.g., GDP). By identifying the products or sectors that present the most strengths and potential to increase the economic development of Paraguay [50], the government from our analysis could identify the accumulative revenues from electricity exports to Brazil and decide accordingly to use those to boost the economic growth of different sectors.

Another techno-economic analysis focused on providing recommendations for Paraguay on a high level hydro based sustainable development strategy with the following pillars: (1) institutional reform and technical improvements of the domestic electricity sector, (2) drafting an industrial strategy based on Paraguay´s comparative advantages and reliable access to clean energy at competitive prices, (3) more favorable and fairer pricing on Itaipu´s sales to Brazil, (4) devising a plan to transition to a green economy and (5) ensuring that revenue collection and management systems are efficient to fund this strategy. This report primarily focuses on Paraguay's electricity sector and is conducted following a qualitative research method based on historical country data and planning strategies to reach conclusions. It can provide an overview of Paraguay's most critical comparative advantages and the barriers holding back sustainable development [52]. One of those is the problems faced by the electricity sector outlined in the report (e.g. low cost of generation, the tariffs, significant reliability constraints with frequent outages, the relatively high transmission and distribution losses) and the lack of coordination due to institutions during the planning phase for future investments among the stakeholders. The authors also believe that Paraguay has not received a fair price for its exported electricity to Brazil. Our study considers those challenges in the electricity supply system as input parameters in the OSeMOSYS model (Sects. 2.1 and 2.2). It shows the government's implications of different electricity demand scenarios and export prices to Brazil, following both a quantitative and qualitative approach to develop appropriate strategic energy planning.

Our study is a novel application since a similar analysis for Paraguay, using a cost-optimization modeling framework for long-term energy planning (OSeMOSYS) considering different electricity demand projections associated with electricity export prices to Brazil for the period 2018–2040, has not been conducted before. Furthermore, the modeling outputs and specifically the electricity exports from Itaipu to Brazil were soft-linked with an input–output project finance model to identify the annual revenues from electricity exports for each scenario missing from the current literature. The main topics analyzed in our work are the followings:

- Strategic energy planning using a cost-optimization modeling framework for long-term energy planning could help define cost-optimal future power plant investments to cover Paraguay´s future electricity needs.
- What are the implications for Paraguay´s national revenues and security of supply of different hydroelectric power export regimes?
- Insights for Paraguay's government on energy transition (e.g. capacity, generation mix, electricity exports) and comparing the government's revenues by setting specific electricity export prices to Brazil to boost the country´s economy.
- Analysis of trade-offs between electricity demand growth and export prices to Brazil.

## Methodology

In this section, we describe the development of the electricity supply system model of Paraguay using the Open Source energy MOdelling SYstem (OSeMOSYS) [48] tool. We present the model structure, in terms of power generation technologies, resources, fuel prices, trade links, and electricity demands, as well as the key assumptions of the analysis. The methodology used in this study can be applied to any country and not only to Paraguay, in what refers to the development of the electricity systems model. This study is a novel application since a similar analysis for Paraguay has not been conducted before. The authors collected country-specific data related to the electricity supply system of Paraguay and processed it to be used as inputs to the OSeMOSYS model. The model outputs associated with the project finance of Itaipu (e.g., electricity exports to Brazil from Itaipu) were used to define the annual revenues for the government of Paraguay and Itaipu. The scenarios explored in the analysis of the Paraguay electricity system are also described in this section of the paper.

### OSeMOSYS

The Open Source energy MOdelling SYstem [48] tool is a dynamic, bottom-up, cost optimization tool for medium to long term energy planning. The tool is comparable to long-established energy systems models such as MARKAL/TIMES [53], MESSAGE [54], PRIMES [55], EFOM [56] and POLES [57]. It is one of the open-source tools featured by the OpTIMUS Community [58]. It has been used widely in academic teaching, capacity building for energy planners and in the scientific literature on developing national energy systems models for African [59] and South American [41] countries [49] as well as recently for Europe [60]. OSeMOSYS determines the least-cost mix of technologies and fuels, satisfying the defined energy demand under several technical (e.g., efficiency, availability, capacity), economic (e.g., operational, investment) and environmental (e.g., emissions) constraints. OSeMOSYS provides results associated with the supply of fuels, the capacity of each technology, the emissions, the operational and maintenance costs, the capital investments as well as prices of electricity. The model outcomes of this study can be provided yearly, indicating the future annual investments to cover the country´s electricity needs over the period 2018–2040. The model considers only the electricity supply system of the country.

### Model structure

The electricity supply system (Supplementary Fig. 3) in this study includes the fuel supply technologies, the power generation technologies, the transmission and distribution network as well as the end-users demand. Each of the technology groups is characterized by economic, technical and environmental parameters, which are defined by the user. The energy resource prices (Supplementary Table 3), the current and future installed capacity as well as the techno-economic parameters for the power generation technologies are based on international and national estimates. The transmission and distribution losses (Supplementary Table 4), as well as the cross-border electricity interconnection projects (Supplementary Table 5), are based on similar sources. The losses in the T&D network are assumed to decrease gradually throughout the modeling period in response to national policies. Existing and future energy policies of the country, such as efficiency improvements in the transmission and distribution network as well as future power plant investments, have been incorporated into the model by adding “constraints” [7]. The electricity demand is disaggregated into the following sectors: commercial, industrial and residential. The electricity demand projections in each one of the scenarios are based on the country´s socio-economic parameters. All parameters considered in the modeling framework are time-dependent.

### Key assumptions

The following assumptions have been made for this analysis:

- The real discount rate is 4%.
- The monetary unit is USD.
- The timeframe of the model reported is from 2018 to 2040, with yearly basis simulations. The period 2040–2045 is added to prevent the “edge effects” as they are distorted by the model, considering that as the ‘end-of-time’.
- In order to capture the key features of electricity demand load pattern, each year has been divided into three seasons, winter (Apr.–Sept.), summer (Dec.–Feb.) and spring (March, Oct., Nov.), and each season in three-day types, namely “day”, “peak”, “night”.
- Existing/committed power plant projects were forced into the model solution. Those are identified as “OPR”: commercial operation, and as “CON”: under construction, physical site work is underway. After that the model may invest in any power plant, identified as a candidate for the system (status “PLN”) (Supplementary Table 1).
- A reserve margin of 20% was considered in line with national policy [7].
- A constant electricity export price was assumed for electricity exports from Paraguay to Argentina, as this is the baseline against which the Itaipu treaty negotiations are likely to be compared. Particular protocols of electricity exchange with neighboring countries considered [10].
- The electricity export price of Itaipu consists of the sum of both the Paraguayan energy cession rate and Itaipu´s average electricity generation cost [10].
- No new future electricity trade flows are considered except the current ones [61].

This study was conducted following several capacity-building activities with local stakeholders and involved institutions in Asuncion, Paraguay, in 2017–2018 [62]. This analysis indicated as fundamental for Paraguay´s electricity expansion plan by government officials. To do this study, a bottom-up open-source cost optimization model for medium to long term energy planning, such as OSeMOSYS, had to be considered.

### Scenarios

**Fig. 1:**
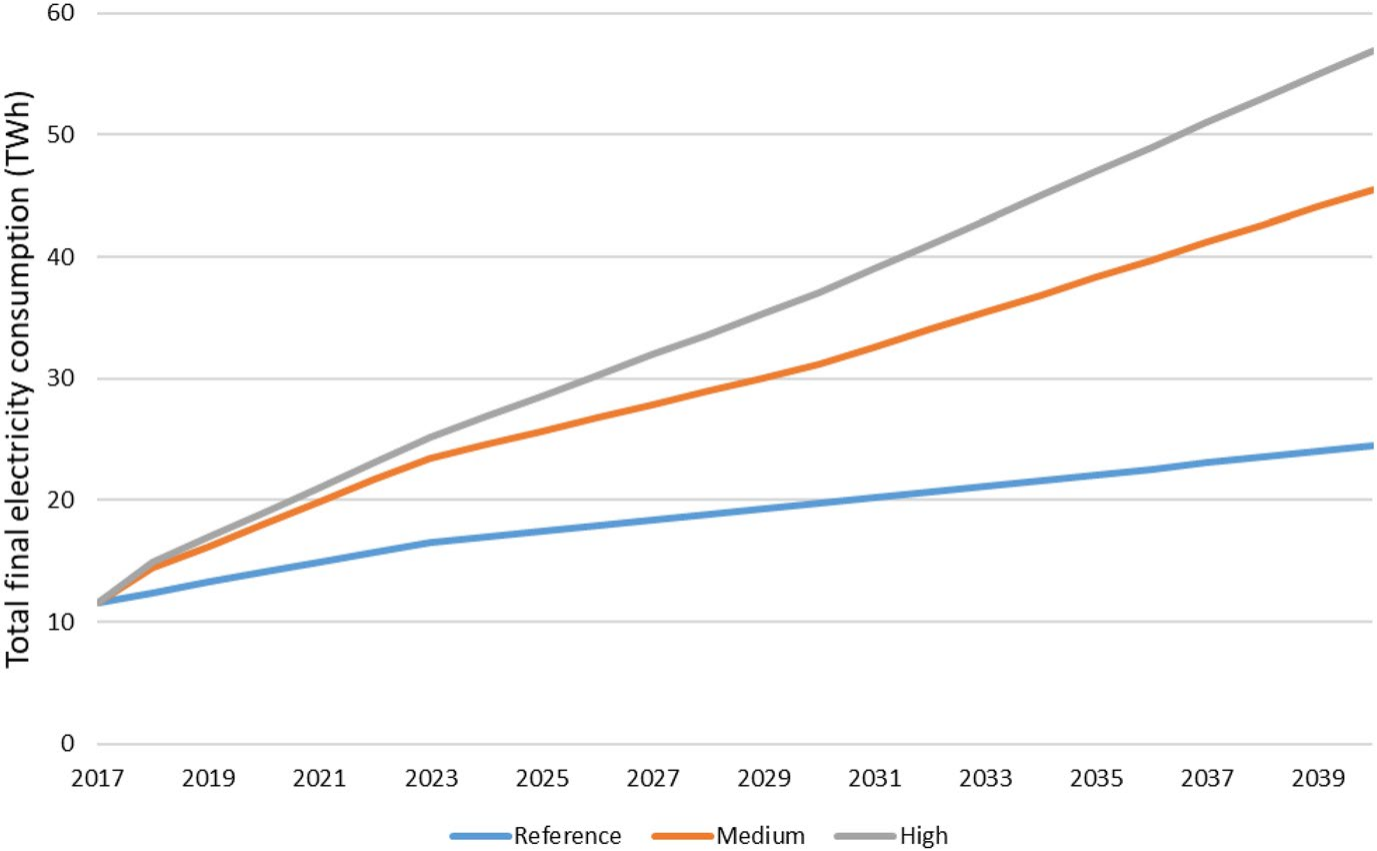
Final electricity consumption per electricity demand scenarios (TWh)

The development of energy scenarios provide insights on plausible changes in the energy system to achieve certain energy, climate and environmental targets. The scenarios investigated in this study consider three different electricity demand projections by 2040 to define the investments needed to satisfy future electricity demand (Fig. 1). On top of that, for each one of the electricity demand trends above, different electricity export price cases associated with the Itaipu plant were conducted. The objective of these scenarios are to assess the possible total profits for Paraguay under different electricity export prices of Itaipu. The scenarios take into account conservative possible outcomes to study for the upcoming negotiations of the Itaipu Treaty. As the debt contracted to construct the Itaipu dam will be paid off by 2023, Itaipu´s finances will significantly improve and the agreed electricity export price will affect Paraguay´s economy [10]. The analysis can provide an overview of the implications of the renegotiation of the Itaipu, Treaty (Annex C, [9]) in 2023, considering different electricity demand outcomes. A detailed breakdown of the electricity demand by sector and scenario for the period 2017–2040 is provided in Supplementary Fig. 1. The different electricity export prices studied in each scenario for the period 2017–2040 are presented in Table 2. The characteristic features of the scenarios are described below (Table 3):

**Table 2:** Summary of the scenarios

	Electricity demand scenarios		
	Reference	Medium	High
	Average growth rate: 3.1%	Average growth rate: 5.38%	Average growth rate: 8.9% (2018–2025); 4.4% (2025–2040)
Export price cases			
ISC1			
Itaipu rate	⇔	⇔	⇔
Cession rate	⇔	⇔	⇔
ISC2			
Itaipu rate	⇓ 60%	⇓ 60%	⇓ 60%
Cession rate	⇔	⇔	⇔
ISC3			
Itaipu rate	⇓ 60%	⇓ 60%	⇓ 60%
Cession rate	⇑	⇑	⇑
ISC4			
Itaipu rate	⇓ 30%	⇓ 30%	⇓ 30%
Cession rate	⇑	⇑	⇑

Changes to the cession rate of Itaipu, in the export price scenarios, are applied from 2023 onwards

**Table 3:** Electricity export price of Itaipu among the electricity demand scenarios the period 2017–2040 [10]

Case	2017–2022	2023	2028	2033	2038	2040
ISC.1						
Average Itaipu rate (USD/MWh)	35.65	35.65	35.65	35.65	35.65	35.65
Cession rate (USD/MWh)	9.579	9.579	9.579	9.579	9.579	9.579
Total	45.23	45.23	45.23	45.23	45.23	45.23
ISC.2						
Average Itaipu rate (USD/MWh)	35.65	14.26	14.26	14.26	14.26	14.26
Cession rate (USD/MWh)	9.579	9.579	9.579	9.579	9.579	9.579
Total	45.23	23.84	23.84	23.84	23.84	23.84
ISC.3						
Average Itaipu rate (USD/MWh)	35.65	14.26	14.26	14.26	14.26	14.26
Cession rate (USD/MWh)	9.579	41.85	33.55	34.59	35.77	36.36
Total	45.23	56.11	47.81	48.85	50.03	50.62
ISC.4						
Average Itaipu rate (USD/MWh)	35.65	24.96	24.96	24.96	24.96	24.96
Cession rate (USD/MWh)	9.579	25.71	21.56	22.09	22.67	22.97
Total	45.23	50.67	46.52	47.04	47.63	47.92

Modern tools for the energy scenario development provide a good basis for the estimates of the required changes in the energy system to achieve certain climate and environmental targets.

- *Reference scenario (BAU)* the evolution of the electricity demand is considered conservative, with an average growth rate of 3.1%. The projections are derived from the report “Elaboración de la prospectiva energética de la República de Paraguay 2013–2040” based on economic growth (GDP growth rate 3.04%) and the evolution of the population (growth rate of 1.21%) by 2040. The productive structure of the country remains at a similar level as the base year, with a high degree of dependence on the agricultural sector, preventing at some level the effect of climate variability. The industrial sector evolves as in previous years [63].
- *Medium Electricity Demand scenario (MED)* the average growth rate of the electricity demand is 5.38%. The population growth is similar to the base scenario, while the economic growth is higher (GDP growth rate 5.16%). The industrial sector increases its competitiveness in the country´s activity level. The projections are taken from the report “Elaboración de la prospectiva energética de la República de Paraguay 2013–2040” [63].
- *High Electricity Demand scenario (HED)* it is considered the penetration of high electro-intensive industries in the country. The average growth rate of the electricity demand is 8.9% until 2025 (high demand growth rate), considering a more aggressive evolution in the industrial sector and 4.4% for 2025–2040 (conservative growth rate). The projections are derived from the report “Plan Maestro de Generación; Periodo 2016–2025” [7].
- *Itaipu—constant electricity export price (ISC.1)* the electricity export price of Itaipu is maintained to create a fund to mainly be used to increase investments in the electricity sector as well as the electricity demand of the industrial sector.
- *Itaipu—a decrease of 60% of the electricity export price (ISC.2)* the average generation cost of Itaipu drops 60% to balance the accounts, because that is the amount that stands for the debt payments, which are finishing in 2023. The cession rate of Paraguayan electricity remains constant [10]. This change will ultimately reward the country that consumes more power.
- *Itaipu—an increase in the electricity export price (ISC.3)* the average generation cost of Itaipu decreases by 60% to balance the accounts. The cession rate of Paraguayan electricity is augmented to compensate for the loss of the value of Paraguayan energy sold to Brazil due to the decrease of the Itaipu rate [10].
- *Itaipu—an increase in the electricity export price (ISC.4)* the average generation cost of Itaipu drops only 30% to create a fund to increase investments in the electricity sector as well as the electricity demand of the industrial sector. The cession rate of Paraguayan electricity is augmented to compensate for the lost value of Paraguayan power sold to Brazil due to the Itaipu rate decrease. The 30% decrease is an assumption in case the government decides to increase the cost again to compensate for the previous debt payments and choose to make an investment fund.

## Results and discussion

### Overview of the results of the reference scenario

In the *Reference—ISC.1 scenario,* the power grid of Paraguay continues to be predominately reliant (99%) on hydro resources in the future. The electricity needs of Paraguay increase from 12.42 TWh in 2018 to 24.40 TWh in 2040. Thus, the existing capacity of the country´s energy system increases from 8.84 GW in 2018, to 11.5 GW in 2026 and 11.65 GW in 2040 to cover the local electricity demand and export the excess electricity. The significant addition of capacity occurs from the hydropower plants Ana Cua (0.27 GW), Ita Cora Itati (0.80 GW) and Corpus Christi (1.44 GW), which collectively have a capacity of 2.51 GW in 2026. Those investments are potential upgrades to Yacyreta´s existing installed capacity (1.60 GW), which could provide electricity exports to Argentina and cover the local energy needs. Model results support the investment plan of the government for the two-stage expansion of Rio Acaray´s plant. The existing installed capacity (0.21 GW) is expanded by 0.065 GW in 2030 and 0.075 GW in 2035 [7]. Also, investments in small hydropower plants, hereinafter referred to as PCH (an abbreviation of the Spanish equivalent "Pequeñas Centrales Hidroelétricas") (0.196 GW) are held in 2024 to provide electricity to the local grid. Fossil fuel plants´ are gradually phased out by 2038.

The estimated electricity export price of Itaipu and Yacyreta affects the future installation of the power plants mentioned above. Thus, the new investments are held only if those are financially attractive solutions and their generation costs are lower than the estimated electricity export prices. In this case, the country exports most of its generated electricity (75% in 2014) to Argentina and Brazil, presenting an overcapacity of the power system.

**Fig. 2:**
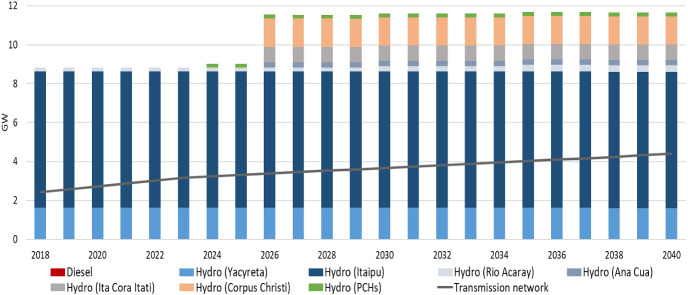
Total installed capacity by technology (GW) in the Reference—ISC.1 scenario

Under this scenario, transmission capacity would need to increase to accommodate the 2 GW of extra generation capacity (Fig. 2).

**Fig. 3:**
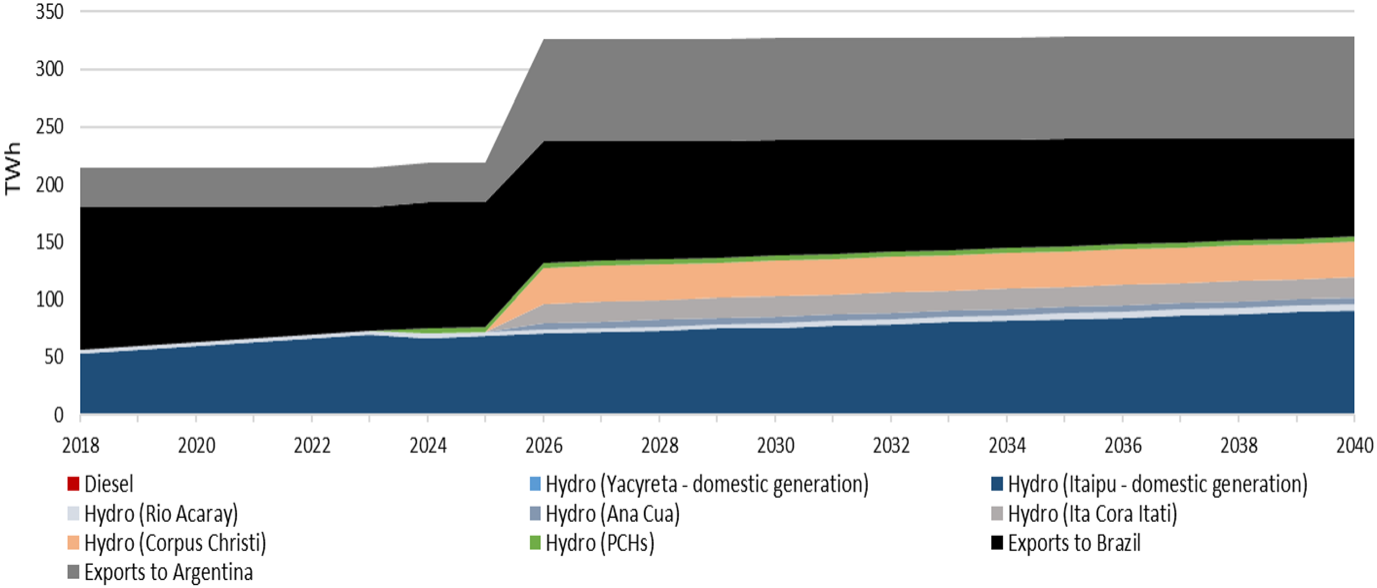
Electricity generation mix by technology (TWh) in the Reference—ISC.1 scenario

The electricity generation in Paraguay increases by approximately 1.5 times by 2040 (91 TWh) compared to 2018 (60 TWh) in the *Reference—ISC.1 scenario*. Hydropower continues to be a significant part of the power mix. The gradual entry of small PCH plants in 2024 increases the electricity supply by 1.22 TWh while the expansion of the power sector on investing in Ana Cua, Ita Cora Itati and Corpus Christi in 2026 will collectively supply electricity of 14.93 TWh annually by 2040. As a result, the electricity exports to Brazil gradually decrease from 34 TWh in 2018 to 24 TWh by 2040 and Argentina increase from 10 TWh in 2018 to 24 TWh by 2040 (Fig. 3). This transition is because the electricity export price of Yacyreta will be higher than Itaipu supplying less electricity to the national grid and decreasing the overall system costs.

### Analysis of trade-offs between electricity demand growth and export prices

#### Implications on generation capacity

As the electricity demand increases among the scenarios (Reference, Medium, High), new hydropower plants are installed (Rio Acaray, Ana Cua, Ita Cora Itati, Corpus Christi, PCHs, new hydropower plant) in different years of the modeling period. Rio Acaray increases its total capacity in 2030 (275 MW) and 2035 (350 MW). Ana Cua is coming into the power system in 2026, adding a capacity of 270 MW while Ita Cora Itati in 2026 (800 MW). Similarly, Corpus Christi is installed in 2026, increasing the total installed capacity by 1.4375 GW. The installed capacity by technology and scenario for the different years of the modeling period can be found in Supplementary Figs. 5–8. One of the factors that affect the total installed capacity and the installation year of the new power plants is the electricity export price of Itaipu and how it affects the electricity exports of the country. We explain this effect in the following paragraphs.

As the electricity export price for Itaipu (ISC.1) remains constant, any differences in the generation capacity are not observed among the different demand scenarios since it is not cost-optimal to invest in new power plants to increase the electricity exports. Nevertheless, as the average generation cost of Itaipu decreases by 60% (ISC.2) to balance the accounts, less capacity is required in the Reference and Medium demand scenarios (11.52 GW, 2040) compared to the High demand scenario (11.65GW, 2040). The noticeable differences among the scenarios are due to the penetration of the PCHs plants into the power system in different years. In the Reference and Medium demand scenario, a capacity of 38.46 MW of PCHs plants come in 2020, while in the High demand scenario, a capacity of 196.49 MW of those power plants operates in 2034.

In the *ISC.3 case*, considering the increase in the electricity export price of Itaipu, in the Reference scenario, the installed capacity increases to 11.78 GW in 2040. A new hydropower plant (130 MW) comes into the power system in the last years of the modeling period (2039–2040). In contrast, in the Medium and High demand scenarios, under the ISC.3 case, the installed capacity increases to 13.65 GW in 2040. The same new hydropower plant comes into the power system in earlier years (2027). In the Medium demand scenario, the installation of the new hydropower plant adds a capacity of 1.24 GW in 2027, reaching to 2 GW in 2031, while in the High demand scenario increases by 2 GW in 2027.

Similarly, in the *ISC.4 case*, in the Reference scenario, the total installed capacity reaches to 11.78 GW in 2040. As in the previous scenario, the higher electricity export price of Itaipu leads to an increase in the installed capacity in the Medium and High demand scenarios reaching 13.65 GW in 2040. The only difference in both the Medium and High demand scenario, is the installation year of the new hydropower plant, which is installed in 2037, adding a capacity of 2 GW.

The variation in the installation year of the new hydropower plant is due to the estimated electricity export price of Itaipu that reaches approximately the same level between the ISC.3 and ISC.4 cases in different years to financially support the new plant and decrease the overall system´s costs. By increasing the generation capacity in the power system, the country has the option to increase its electricity exports to the neighboring countries. As can be seen in Fig. 9, the average electricity cost of generating electricity for the new hydropower plants in the ISC.3 and ISC.4 cases is lower than the electricity export price of Itaipu.

#### Implications on the generation mix and electricity exports

Under the *ISC.1 case,* the power generation throughout the modeling period increases to 42 TWh in 2040 in the Reference scenario compared to 68 TWh in the Medium and the High demand scenarios in the same year (Supplementary Fig. 9). However, the electricity generation mix changes among the scenarios affected primarily by both electricity exports and local electricity demand. In the Medium and High demand scenarios, the industrial penetration and the improvement of socio-economic conditions increase the domestic electricity demand. In the above scenarios, this transformation leads to a substantial decrease in the country´s overall electricity exports by approximately 28% and 41% correspondingly during 2018–2040 compared to the Reference scenario one. In this case (ISC.1), the electricity export price of Itaipu is assumed to be lower than the Yacyreta´s one, so it is not profitable to increase the levels of electricity exports to Brazil. Also, sufficient reserve generation capacity is needed to be maintained to cover peak demand.

**Fig. 4:**
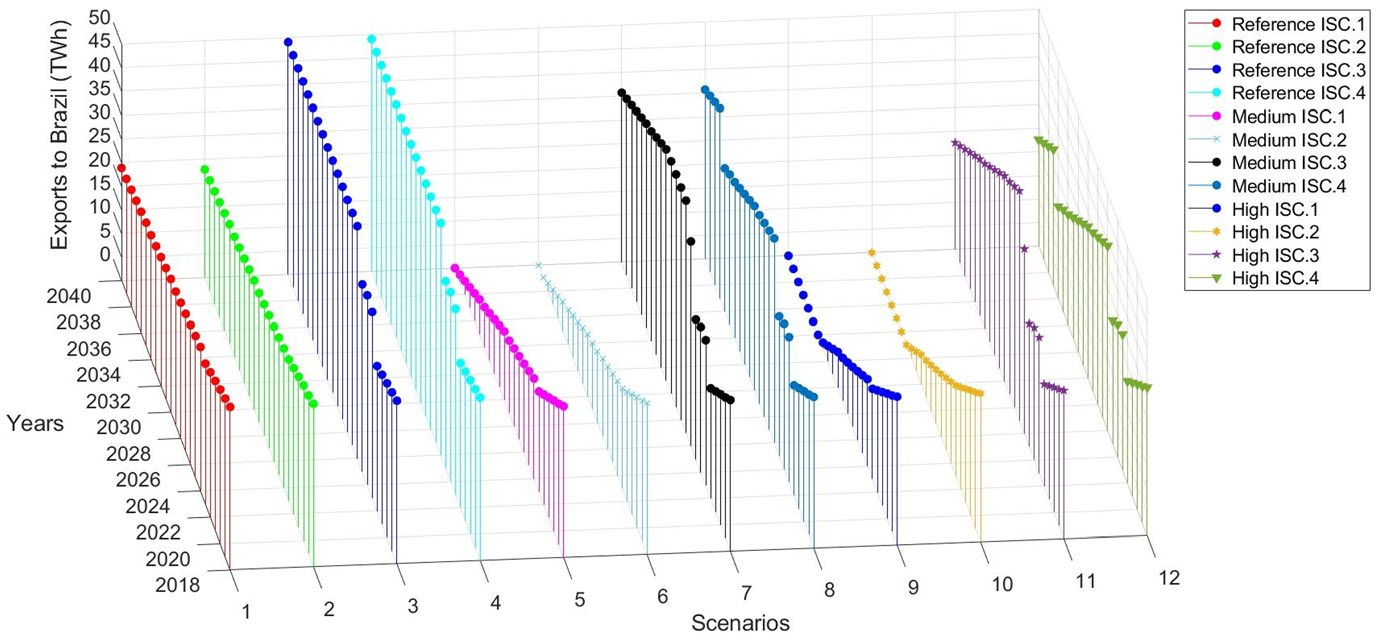
Electricity exports to Brazil among the different demand scenarios combined with the electricity export price of Itaipu

**Fig. 5:**
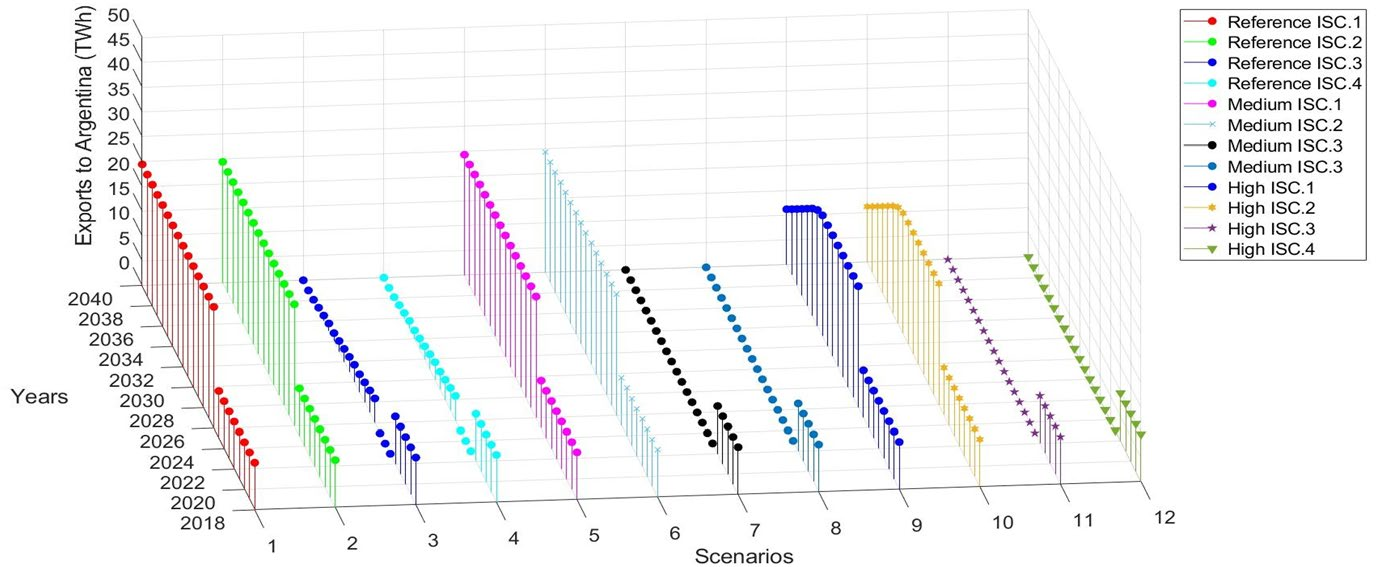
Electricity exports to Argentina among the different demand scenarios combined with the electricity export price of Itaipu

The results show that in the Reference scenario, the electricity exports to Brazil decrease from 34 TWh in 2018 to 24 TWh in 2040. In the Medium and High demand scenarios, the exports to Brazil reach gradually to 0 TWh in 2040 (Fig. 4). It is interesting to notice that in this scenario, the higher electricity export price of Yacyreta compared to Itaipu leads to higher electricity exports to Argentina while shifting Itaipu´s generation mainly to cover the domestic demand. Ana Cua, Ita Cora Itati and Rio Acaray supply electricity in a total of 14.94 TWh annually and small PCHs plants 1.22 TWh annually starting operation in different years. On the other hand, the electricity exports to Argentina increase in the period 2026–2036 compared to the previous decade, mainly due to the increase of the power generation capacity. Specifically, in the Reference and Medium demand scenarios, the electricity exports to Argentina increase from 10 TWh in 2018 to 24 TWh in 2040. However, in the High demand scenario, the electricity exports to Argentina increase to 24 TWh in 2026 and then gradually decrease to 11 TWh in 2040 (Fig. 5). This trend is because the electricity consumption per capita in the country is very low. Thus, as the domestic electricity demand increases across the scenarios, overall electricity exports are decreasing in this electricity export price scenario.

Under the *ISC.2 case*, in the Reference scenario, the domestic electricity supply reaches approximately 43 TWh in 2040 while in the Medium and High demand scenarios reaches 67 TWh correspondingly (Supplementary Fig. 10). Besides, in the Reference scenario, the total exports increase to around 47 TWh compared to the Medium (24 TWh) and High (11 TWh) demand scenarios by 2040. Similarly to the ISC.1 case, the electricity export price of Itaipu is lower than in the Yacyreta one in the future, leading to lower electricity exports to Brazil among the scenarios. In the Reference scenario, the electricity exports to Brazil decrease by 32% (reaching 23 TWh) by 2040, while in the other two scenarios decrease, reaching gradually to 0 TWh in 2040. The rest of the total electricity exports are in Argentina. Furthermore, in the High demand scenario, the small PCHs plants provide electricity domestically 1.22 TWh on an annual basis to increase the electricity exports to Argentina around 2.6 times compared to the previous years starting from 2026.

Under the *ISC.3 case*, in the Reference scenario, the domestic electricity supply reaches 28 TWh in 2040 compared to 52 TWh (Medium) and 65 TWh (High) in the same year. Moreover, in each of the scenarios, the electricity exports to Argentina gradually decrease, reaching 0 TWh by 2040. However, the electricity exports to Brazil increase in the Reference (44%) and Medium (3%) scenario by 2040 while in the High (− 32%) decrease. The electricity export of Itaipu is higher than Yacyreta, leading the country to increase its electricity exports to Brazil compared to Argentina´s one in each of the scenarios by 2040. Besides, the future annual power generation of Rio Acaray, Ana Cua, Ita Cora Itati and Corpus Christi is similar among the scenarios throughout the modeling period. Lastly, a new hydropower plant is installed in different years among the scenarios (Supplementary Fig. 11).

In the *ISC.4 case*, a similar observation, as in the *ISC.3 case*, applies in each of the demand scenarios. The overall electricity generation increases in the Reference (77 TWh), Medium (88 TWh) and High (88 TWh) demand scenarios by 2040. The electricity exports are only to Brazil, wherein the Reference scenario, they constitute around 64% by 2040 while in the Medium (41%) and High (26%) demand scenarios, less in the same year (Supplementary Fig. 12).

#### Total profits for Paraguay under the electricity export price of Itaipu

In this section, we analyze the implications of the different demand levels on the electricity sector of Paraguay and the country´s economy, focusing on the Itaipu power plant, under the different demand and electricity export price scenarios. As the Itaipu debt is expected to be paid by 2023, the government could use the profits from the electricity exports to Brazil from Itaipu to boost Paraguay´s economy.

In the *Reference scenario*, under *ISC.1 case* the country`s total profits from Itaipu increase from 0.39 BUSD in 2018 to 1.31 BUSD by 2040. While in each of the cases *ISC.2, ISC.3, ISC.4*, the total profits reach 0.26 BUSD, 1.82 BUSD and 1.69 BUSD respectively in the same year. The total earnings for the government from Itaipu are following the electricity export price between Itaipu and Brazil. As the electricity export price to Brazil is increasing and being higher than the electricity export price to Argentina, Itaipu increases its electricity exports to Brazil. Besides, as it has been analyzed in the previous section, the penetration of the new hydropower plans, mainly the following years 2023, 2026, further increases the electricity exports to Brazil, mainly in *ISC.3* and *ISC.4* cases (Supplementary Fig. 13).

**Fig. 6:**
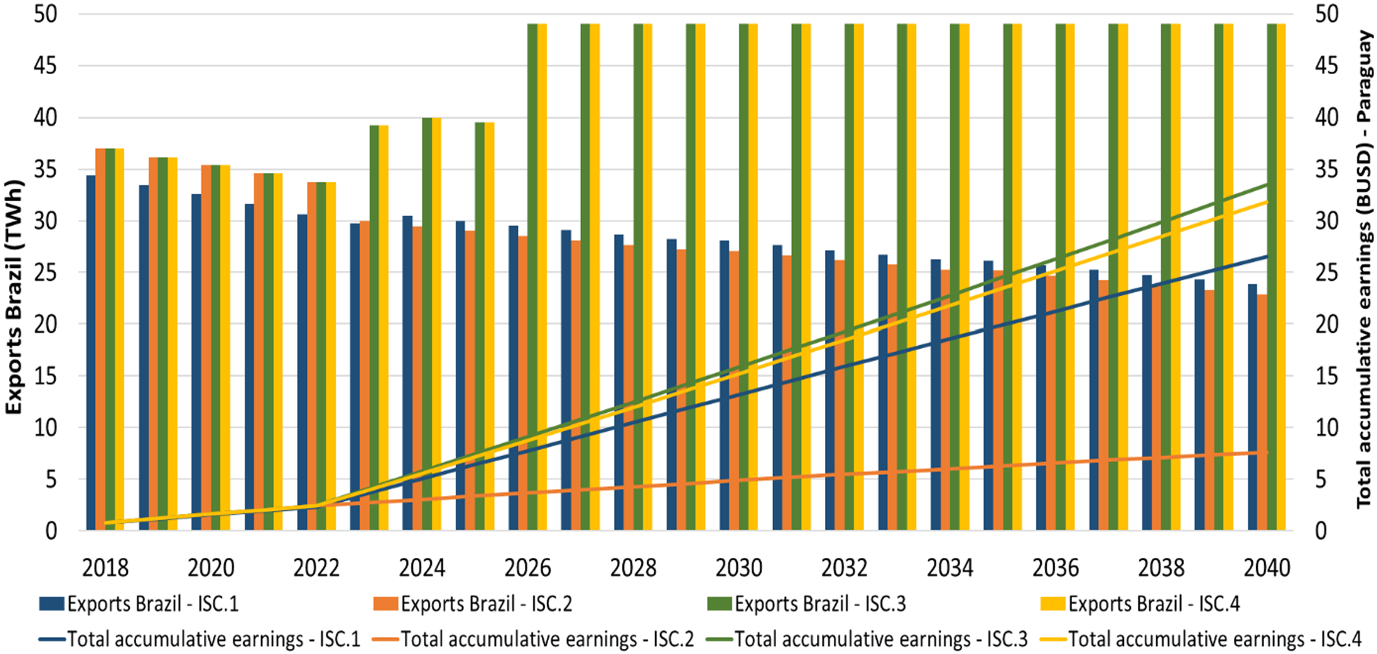
Reference scenario—exports to Brazil (TWh) vs total accumulative earnings (BUSD) for Paraguay from Itaipu

In the Reference scenario, the total accumulative earnings (in BUSD) for Paraguay from Itaipu (*ISC.1–4)*, as a function of the electricity exports to Brazil, are illustrated in Fig. 6. The total accumulative earnings increase from 0.8 BUSD in 2018 to 27 BUSD (ISC.1), 8 BUSD (ISC.2), 33 BUSD (ISC.3) and 32 BUSD (ISC.4) in 2040 respectively among the scenarios.

In the *Medium demand* scenario, under the *ISC.1* and *ISC.2* cases, the future electricity exports to Brazil decrease while in the *ISC.3* and *ISC.4* cases increase. Thus, in the first case, the total profits for Paraguay are 1.09 BUSD (*ISC.1*) and 0.04 BUSD (*ISC.2)* in 2040, while in the second case are 1.34 BUSD (*ISC.3)* and 1.38 BUSD (ISC.4) accordingly in the same year (Supplementary Fig. 14).

**Fig. 7:**
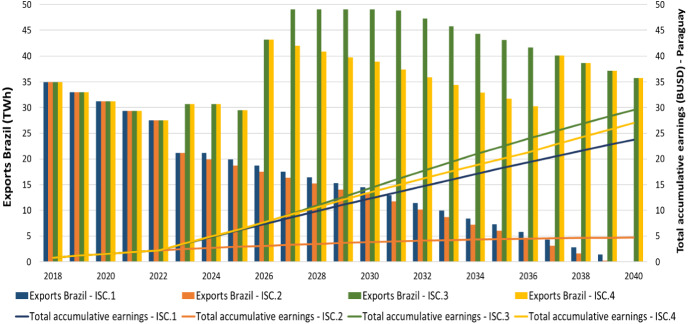
Medium demand scenario—exports to Brazil (TWh) vs total accumulative earnings (BUSD) for Paraguay from Itaipu

In the same demand scenario, the total accumulative earnings for the government of Paraguay reach to 23.77 BUSD (*ISC.1)*, 4.70 BUSD (*ISC.2)*, 29.52 BUSD (*ISC.3)* and 26.99 BUSD (*ISC.4)* in 2040 respectively. The total earnings in each of the scenarios are lower than in the Reference scenario, since the domestic electricity demand is higher (Fig. 7).

In the High demand scenario, the total annual profits for the government of Paraguay reach 1.09 BUSD (*ISC.1*), 0.04 BUSD (*ISC.2*), 0.86 BUSD (*ISC.3*) and 1.08 BUSD (*ISC.4*) in 2040 respectively. It should be noted how the total annual profits in each one of the scenarios differ, starting from 2023 onwards, as the electricity export price fluctuates and new power plants are installed in the future (Supplementary Fig. 15).

**Fig. 8:**
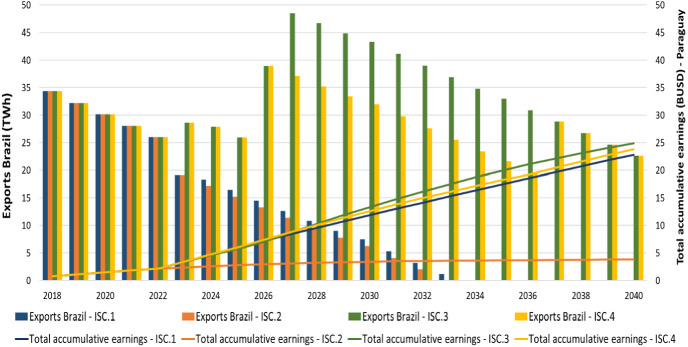
High demand scenario – Exports to Brazil (TWh) vs Total accumulative earnings (BUSD) for Paraguay from Itaipu

In the *High demand* scenario, the total accumulative earnings reach among the scenarios to 22.85 BUSD (*ISC.1*), 3.85 BUSD (*ISC.2*), 24.89 BUSD (*ISC.3*) and 23.80 BUSD (*ISC.4*) in 2040, correspondingly Fig. 8.

**Fig. 9:**
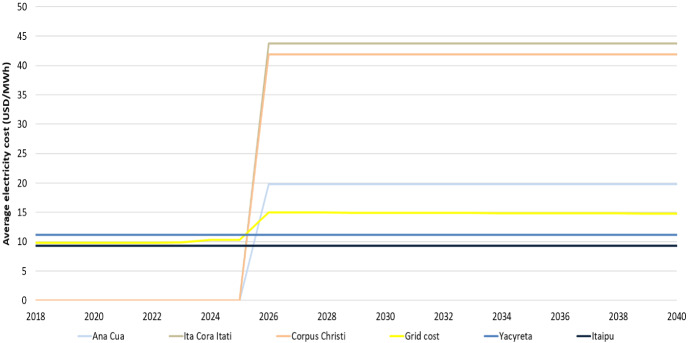
Average electricity cost of generating electricity (USD/MWh)

The estimated average electricity cost of generating electricity for the different power plants in Paraguay is illustrated below (Fig. 9). As the electricity export price of Itaipu changes throughout the years (starting from 2023), it affects the installation of the new power plants based on their average cost of generating electricity.

## Discussion

This study provides insights for Paraguay on long-term electricity planning, considering future investments in the power system and compares the revenues for the government by setting specific electricity export prices to boost the country´s economy. The electricity demand projections analyzed emphasize the importance of strategic energy planning. Even though Paraguay has overcapacity in the power system to supply domestic electricity demand, the generation capacity needs to be expanded in the future. The additional capacity is essential to keep the electricity export levels at a sufficiently high level while maintaining a suitable reserve margin to ensure system reliability. Paraguay has a high renewable energy potential to invest. However, the cheap electricity produced by the Itaipu and Yacyreta hydropower plants, and the overcapacity of the power system, makes this alternative less profitable at some level (Fig. 9). The model results support the investment plan of the government in the power sector [6,28,39]. However, the future implementation of some hydropower projects (Ana Cua, Ita Cora Itati, Corpus Christi) is more challenging because they are binational joint projects and both countries must agree in their approval. Investments on new hydropower plants could ensure revenues in the government to be used for other purposes. Decision-makers must consider the financial viability of the economic potential of investing in a large hydropower project, policies for resettlements, hydrological uncertainties, as well as geopolitical challenges [35]. One of the biggest challenges the government faces nowadays is negotiating the electricity export price of Itaipu to Brazil, which benefits both countries. The negotiation of the terms of the Itaipu Treaty in 2023 will have several implications in the country´s economy in the coming decade. The higher electricity export prices to Brazil from Itaipu (ISC.3 and ISC.4 scenarios), compared to the price agreed until 2022, and Argentina´s current ones could allow the government to increase its revenues further and to improve power infrastructure on a national level. In the opposite case (ISC.1 and ISC.2), it would be profitable to decrease the electricity exports to Brazil from Itaipu and use Itaipu´s generation instead to cover most of the country´s electricity needs and increase the electricity exports to Argentina.

Nevertheless, in the High demand scenario, the country´s electricity exports dramatically decrease by 2040. Paraguay is an electricity hub exporter in South America; thus, any decisions on the country’s national energy plan has a substantial effect on the neighboring countries. The outcomes of this study point out the key investments, trade and policy recommendations as a base for an outlook for power purchase agreements.

In the Reference demand scenario, Paraguay covers its energy needs until 2040, taking into consideration the country´s National Development Plan for 2014–2030 [28]. Also, it maintains its electricity exports to Argentina and Brazil at similar levels compared to 2018 by investing in new hydropower plants, mainly in 2026. On the supply side, the country´s fuel mix continues to be based entirely on renewables. Those results have a positive impact on the country´s economy in the long-term and would include the creation of new income opportunities, jobs and livelihoods [64]. Nevertheless, in the Reference—ISC.1 scenario, the government spends approximately 18.3 billion USD on power plant investments and grid expansion throughout the period 2018–2040. As the national electricity demand increases, the further development of Yacyretá dam is essential in 2026 (Supplementary Figs. 5–8), which is consistent across scenario results.

In the Medium and High demand scenarios, the investments on new power plants and the expansion of the grid correspond to approximately 25.4 billion USD and 31.2 billion USD accordingly for the period 2018–2040. The establishment of a similar electricity export price after 2023 compared to 2018 market levels could allow the government to create a funding scheme available to invest in the country’s economic growth [3]. The government could potentially use these funds to invest in the development of its industrial sector [44], improve its transport infrastructure, which could lead to decreasing fossil fuel imports and enhancing its energy security. Another option could be to subsidize the acquisition of electric stoves contributing to socio-economic development [65]. Moreover, the government could exploit its significant potential for the production of biofuels [66] for national consumption as well as exports boosting the country´s economy following Brazil’s example [67]. The High demand scenario is consistent with higher investments in the energy-intensive industry. Thus, it appears that the country could cope with this—and should actively attract intensive industry—or leverage higher prices from its electricity exports. Following this, in the High demand scenario, the electricity exports are decreasing considerably in the future, by 50% compared to the Reference demand scenario in 2040, despite the planned investments. However, the central management of the grid and infrastructure development is essential. Frequent power shortages can result in significant losses in industry sales and damage equipment. To pursue key policy priorities, the government and significant stakeholders are vital to devote time, capital investments and strong political will.

The scope of this analysis could be extended to considering not only the electricity supply system of the country but other sectors such as transport and residential. Besides, trade links with other neighboring countries (Bolivia, Chile, Uruguay), demand-side management, energy efficiency measures and consumer behavior could also be considered. Moreover, modeling the storage of hydropower plants could provide the flexibility of the operation of hydro and better utilization of this power source. Also, the demand drivers under different scenarios could be further analyzed to capture possible future changes in socio-economic factors (e.g., population, GDP).

The flexibility of operation of hydro and pumped-storage power plants and the variety of ancillary services that they provide to the grid enable better utilization of variable renewable resources and more efficient and reliable operation of the entire power system. A model with cascading hydropower is to prefer to capture all the possibilities with hydropower and hydropower storage. The level of detail to model hydropower storage in OSeMOSYS strongly depends on what the analysts are looking for in their research work [68]. Nevertheless, the policy insights offered by our model and the detail of the analysis (e.g., annual electricity demand split into nine-time slices) do not need to model hydropower with storage explicitly. Furthermore, the location-specific environmental minimum river flows associated with current and future hydropower plants would be required to model storage in detail. The detailed level of these data is missing from the literature.

Future work would also include updating some of the techno-economic parameters extracted from international sources associated with future power generation technologies with local sources. The model developed assuming price-inelastic demand, free competition with no market imperfections and predetermined projections for electricity demand, fuel prices, power generation costs, resource availability and energy policies. Future uncertainties regarding the evolution of those parameters are not modeled. Besides, scenarios are carefully constructed snapshots of the future and the possible ways a sector might develop to draw insights and not predict the future [69]. One of those future uncertainties is the COVID 19 pandemic, which impacted the power sector in terms of both overall demand (decline in the electricity demand) and consumption patterns (generation mix, changes in fuel prices) [70]. The pandemic also affected the country´s economy, forecasting a decline in GDP of 5% in 2020 compared with 4%, which was predicted [71].

The open-source studies as the current one can strengthen the duplication efforts, improve the datasets´ quality by verifying and updating-up to date modeling assumptions and being transparent to overcome criticism [13,72].

Future work could also examine the macro-economic impacts (e.g., exports, revenues, impact upon employment, GDP) associated with the energy sector's evolution using a top-down modeling approach [73–75], [75, p. 3]. However, this approach misses the technical detail of the energy transition, which our study covers. An electricity model for Paraguay, which uses both modeling approaches, could offer broader policy insights associated with the evolution of the economy coupled with the energy sector.

Since the water availability in the Paraná River could significantly affect the country´s power generation mix, a climate change scenario could be examined. The findings of this paper are meaningful for other developing countries of comparable socio-economic characteristics, a (large) hydro-dominated energy system and significant electricity exporters where a similar methodology could be applied to assist in policy-decision making in energy transition (e.g., Brazil [76,77], China [78,79], India [80] and African countries [81]).

## Conclusions

In this study, we applied an open-source cost-optimization modeling system for long-term energy planning (OSeMOSYS) in the development of a model of the electricity system of Paraguay. We used the model to investigate hypothetical futures of electricity from 2018 to 2040. Different scenarios examined related to a combination of varying electricity demands, electricity export prices, and cession rates of Itaipu. The cost-optimal power generation mix of Paraguay under the different scenarios is identified. Also, we estimated the annual revenues for the government of Paraguay and Itaipu through its electricity exports to Brazil. We find that Paraguay needs to expand the capacity of its power system, mainly by investing in hydropower plants, to cover its future electricity needs and sustain national electricity export levels. The country´s overall exports decrease by 50% by 2040 between the high demand scenario and the Reference scenario, assuming the electricity demand in the High demand scenario could approximately double by 2040. The results suggest an investment requirement of 18.3–31.2 billion USD,across the scenarios, in electricity generation infrastructure for the period 2018–2040 to cover future electricity demand. A higher cession rate on electricity exports from Itaipu to Brazil after 2023 (ISC.3–4) compared to the previous levels leads Itaipu to increase its electricity exports to Brazil and Yacyreta to swift its electricity generation to cover domestic electricity needs.

## Supplementary Information

Below is the link to the electronic supplementary material.

Supplementary file1 (DOCX 1051 KB)

## Data Availability

The dataset used to develop the model as well as the model can be found in the Github repository.
