# Supplementary material for: Implications to the electricity system of Paraguay of different demand scenarios and export prices to Brazil
Source: Energy Syst. 2021 Jan 11;12(4):911–39. doi: 10.1007/s12667-020-00420-w (PMC7797354; doi:10.1007/s12667-020-00420-w)
Supplement: Supplementary file 1 — Supplementary file1 (DOCX 1051 KB) [file 12667_2020_420_MOESM1_ESM.docx]

Supplementary material

Electricity expansion investment Outlook and financial implications for Paraguay and Itaipu under different demand scenarios

I.Pappis et al.

Supplementary Tables

In the following tables 1-2, the techno-economic parameters of the existing and future electricity generation projects are presented.

**Supplementary Table 1.** Existing and future planned electricity generation projects (MW) and economic parameters by technology ([1], , [2]).

| Name of the plant | Capacity (MW) | Technology | Capital Cost ($/kW) | Fixed Cost ($/kW) | Variable cost ($/kWh) | Earliest year | Status |
| --- | --- | --- | --- | --- | --- | --- | --- |
| Bahia Negra | 0.565 | IC - Diesel | - | 20 | - | 1980 | OPR |
| Fuerte Olimpo | 0.5 | IC - Diesel | - | 20 | - | 1980 | OPR |
| Pedro J Caballero | 3 | IC - Diesel | - | 20 | - | 1980 | OPR |
| Puerto Vallemi | 1.7 | IC - Diesel | - | 20 | - | 1980 | OPR |
| Rio Acaray | 0.25 | IC - Diesel | - | 20 | - | 1980 | OPR |
| Salto Del Guaira | 20.875 | IC - Diesel | - | 20 | - | 2013 | OPR |
| San Carlos Ande | 0.08 | IC - Diesel | - | 20 | - | 1980 | OPR |
| Yacyreta | 1600 (Total 3,200 MW, 50% shared with Argentina) | Hydro dam | - | 66.67 | - | 1994 | OPR |
| Itaipu | 7000 (Total 14GW, 50% shared with Brazil) | Hydro dam | - | 25 | 0.0056 | 1984 | OPR |
| Rio Acaray | 45;45;126.2;65;75 | Hydro dam | 400 | 18.69 | - | 1968;1970;1979;2030;2035 | OPR-PLN-PLN |
| Ana Cua | 270 | Hydro dam | 1,229 | 66.67 | - | 2026 | PLN (Binational power plant. This has been added as an upgrade to Yacyreta) |
| Ita Cora Itati | 800 | Hydro dam | 4,643 | 66.67 | - | 2026 | PLN (Binational power plant. This has been added as an upgrade to Yacyreta) |
| Corpus Christi | 1437.5 | Hydro dam | 4,370 | 66.67 | - | 2026 | PLN (Binational power plant. This has been added as an upgrade to Yacyreta) |
| PCH Capiibary Emplazamiento | 5.61 | Hydro (run-of-river) | 7,312 | 20 | - | 2022 | PLN |
| PCH Carapa Emplazamiento | 4.24; 18.27 | Hydro (run-of-river) | 3,882; 3,244 | 10.68; 8.77 | - | 2022; 2024 | PLN |
| PCH Itambey Emplazamiento | 4.81 | Hydro (run-of-river) | 3819 | 10.48 | - | 2024 | PLN |
| PCH Jejui Emplazamiento | 5.3; 9.81; 6.7 | Hydro (run-of-river) | 3,775; 3,496; 3,666 | 10.36; 9.51; 10.03 | - | 2021; 2022; 2024 | PLN |
| PCH Nacunday Emplazamiento | 33.34; 7.5; 53.27 | Hydro (run-of-river) | 3,019; 3,613; 2,857 | 8.12; 9.84; 7.65 | - | 2020; 2023; 2024 | PLN |
| PCH Pirajui Emplazamiento | 8.6 | Hydro (run-of-river) | 3,552 | 9.66 | - | 2024 | PLN |
| PCH Tembey Emplazamiento | 3.58; 2.71; 10.07 | Hydro (run-of-river) | 7,642; 4,107; 3,485 | 21.03; 11.29; 9.47 | - | 2021; 2022; 2023 | PLN |
| PCH Ypane Emplazamiento | 5.12; 14.38; 3.18 | Hydro (run-of-river) | 3,789; 3,829; 4,028 | 10.37; 10.5; 11.13 | - | 2019; 2021; 2022 | PLN |
| Hydropower plant (new) |  | Hydro | 4800 | 73.3 |  | 2025 | PLN |
| Biomass power plant | - | Biomass | 2,332 (2014), 2,277 (2040) | 75 | - | 2025 | PLN |
| Natural Gas power plant | - | NG | 818 | 25 | - | 2032 | PLN |
| Solar (CSP with storage) | - |  | 6,895 (2014), 4,747 (2040) | 260 (2014), 180(2040) | - | 2022 | PLN |
| Solar (CSP without storage) | - |  | 6,000 (2014), 4,053 (2040) | 260 (2014), 180 (2040) | - | 2022 | PLN |
| Solar PV (Commercial) | - |  | 2,287 (2014), 1,154 (2040) | 24 (2014), 20 (2040) | - | 2022 | PLN |
| Solar PV (Residential) | - |  | 2,287 (2014), 1,154 (2040) | 24 (2014), 20 (2040) | - | 2022 | PLN |
| Wind (Centre-North area) | - |  | 1,936 (2014), 1,772 (2040) | 48 (2014), 44 (2040) | - | 2022 | PLN |

**Supplementary Table 2.** Technical parameters for power generating technologies [3], [4].

| Technologies | Efficiency (%) | | | | Avail. factor (%) | Life (Yrs) | Constr. Time (Yrs) | Load factor (%) |
| --- | --- | --- | --- | --- | --- | --- | --- | --- |
|  | **2014** | **2020** | **2030** | **2040** |  |  |  |  |
| Biomass power station | 35 | 35 | 35 | 35 | 60 | 25 | 4 | 70 |
| Diesel power station (ICE) | 38 | 38 | 39 | 41 | 90 | 25 | 2 | 93 |
| HFO power station | 38 | 38 | 39 | 41 | 90 | 25 | 2 | 93 |
| Hydropower plant (new) | 100 | 100 | 100 | 100 | 28 | 50 | 6 | 28 |
| Natural Gas power station | 38 | 38 | 39 | 41 | 90 | 25 | 3 | 93 |
| Wind power station | 100 | 100 | 100 | 100 | 100 | 25 | 2 | Varies |
| Solar Thermal with storage | 100 | 100 | 100 | 100 | 100 | 25 | 3 | Varies |
| Solar Thermal no storage | 100 | 100 | 100 | 100 | 100 | 25 | 3 | Varies |
| Rooftop PV – commercial buildings | 100 | 100 | 100 | 100 | 100 | 20 | 1 | Varies |
| Rooftop PV – residential buildings | 100 | 100 | 100 | 100 | 100 | 20 | 1 | Varies |
| Yacyreta - hydro power plant | 100 | 100 | 100 | 100 | 68 | 80 |  | 68 |
| Itaipu - hydro power plant | 100 | 100 | 100 | 100 | 80 | 80 |  | 85 |
| Rio Acaray – hydro power plant | 100 | 100 | 100 | 100 | 80 | 80 |  | 51 |
| Existing Diesel IC | 50 | 50 | 50 | 50 | 90 | 25 |  | 51 |

The fuel costs assumed for the modelling period 2018-2040 are presented in the following table.

**Supplementary Table 3.** Fuel costs [3], [4].

| USD/GJ | 2018 | 2020 | 2030 | 2040 |
| --- | --- | --- | --- | --- |
| Electricity exports to Brazil | Differs for each scenario | Differs for each scenario | Differs for each scenario | Differs for each scenario |
| Electricity exports to Argentina | 12.6 | 12.6 | 12.6 | 12.6 |
| Electricity imports | 45.00 | 45.00 | 45.00 | 45.00 |
| Heavy Fuel Oil imports | 20.18 | 23.51 | 32.08 | 36.9 |
| Diesel imports | 20.8 | 24.2 | 34 | 38 |
| Natural gas imports | 10.8 | 10.86 | 15.75 | 17.6 |
| Biomass | 1.25 | 1.25 | 1.25 | 1.25 |

The evolution of the transmission and distribution losses in the local grid of Paraguay can be found in the following table.

**Supplementary Table 4.** Losses in the transmission and distribution network [1].

| Losses (%) | 2018 | 2020 | 2030 | 2040 |
| --- | --- | --- | --- | --- |
| Transmission | 4 | 4 | 4 | 4 |
| Distribution | 17 | 16 | 11 | 8 |

The electricity interconnector projects with the neighboring countries are illustrated in the following table.

**Supplementary Table 5.** Cross-border electricity interconnection projects [5].

| Interconnections | MW |
| --- | --- |
| Paraguay - Argentina | 3,000 |
| Paraguay - Brazil | 6,100 |

Supplementary Figures

The following figure illustrates the final electricity demand by scenario.

**Supplementary Figure 1.** Final electricity consumption (TWh) by sector in the Reference, Medium and High demand scenarios [6] [1].

**‘**

The schematic representation of the energy system (RES) of Paraguay, starting from supply to end-user activities, is presented in the figure below.


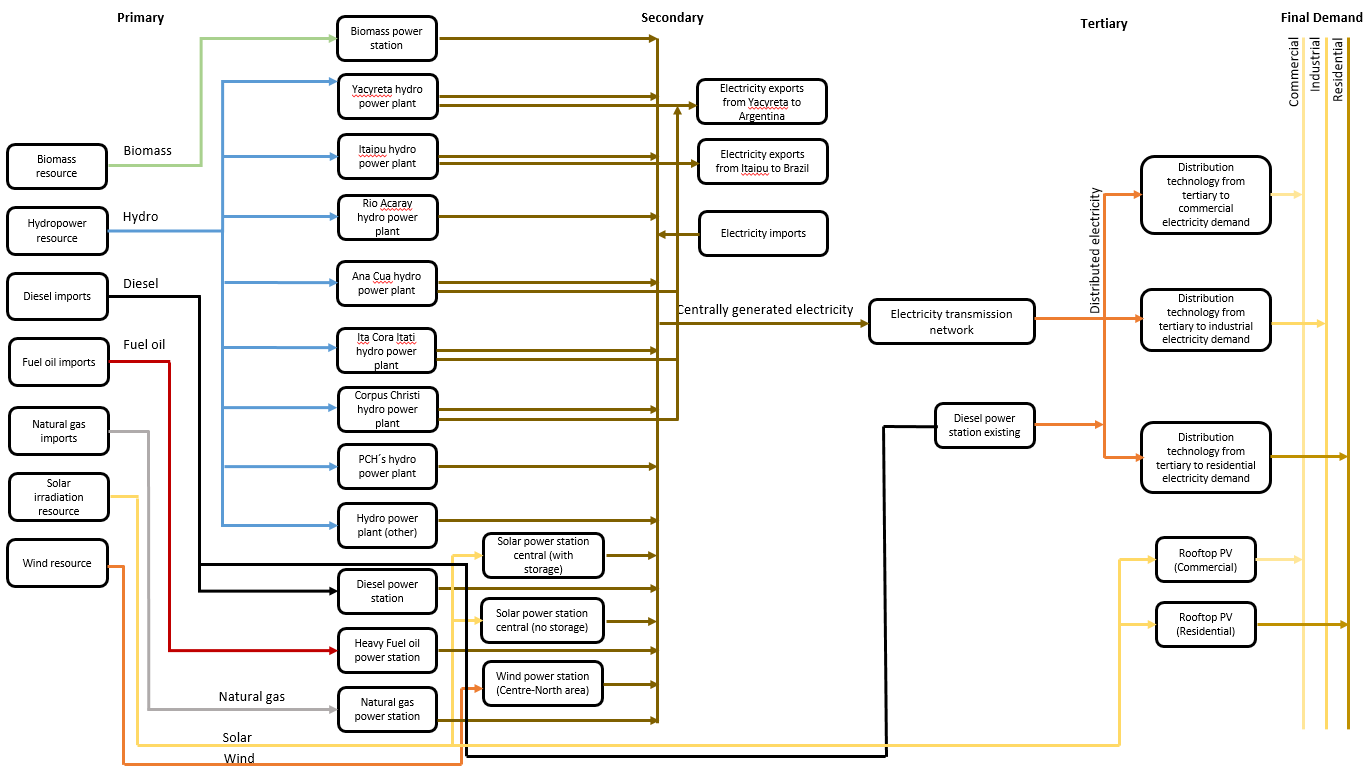


**Supplementary Figure 3.** Reference Energy System (RES).

**Supplementary Figure 4.** Electricity network of Paraguay in 2018 [1].

The total installed capacity by power generation technology under the different demand combined with electricity export price scenarios is presented in the following figures (Figure 5-8).


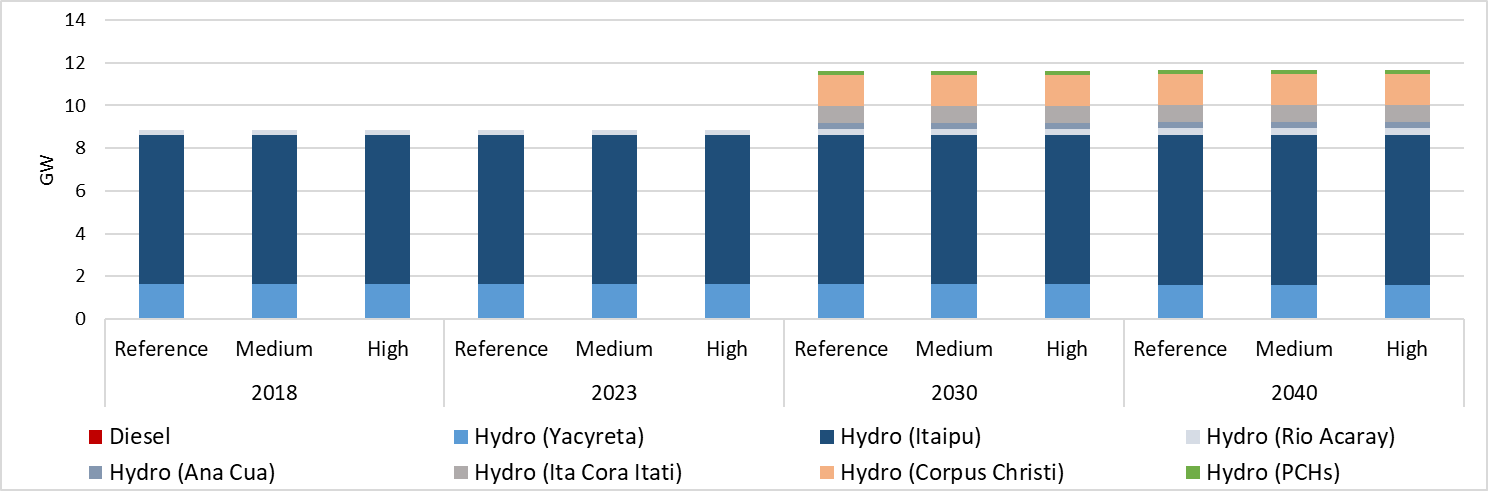


**Supplementary Figure 5.** Total installed capacity by technology (GW) under the different demand – ISC.1 scenarios.


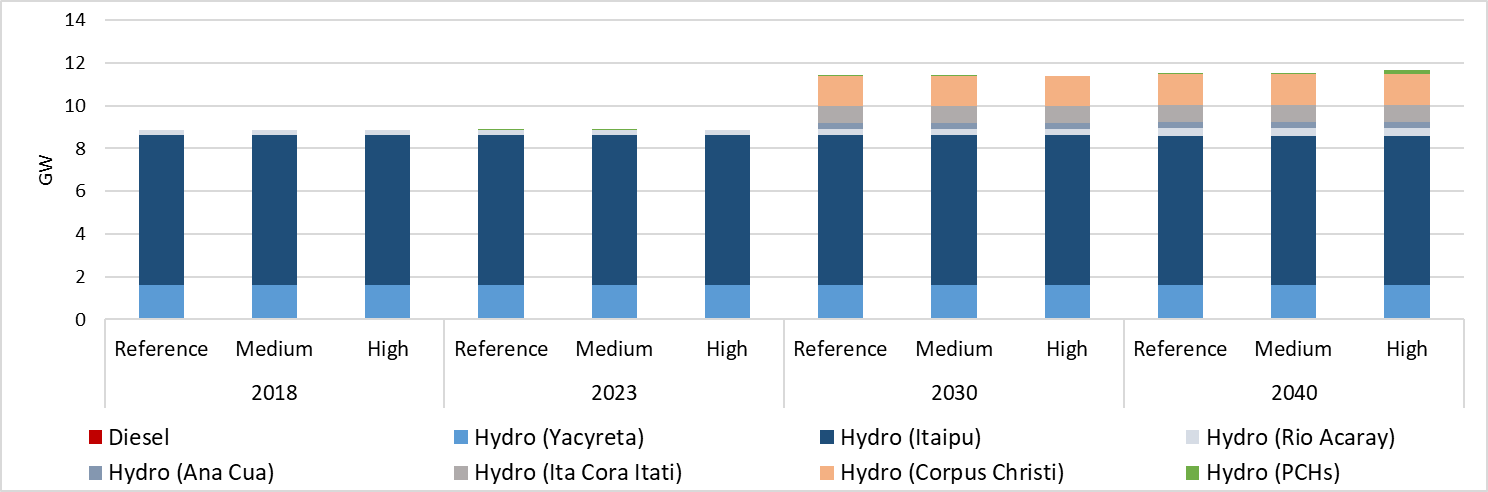


**Supplementary Figure 6.** Total installed capacity by technology (GW) under the different demand – ISC.2 scenarios.


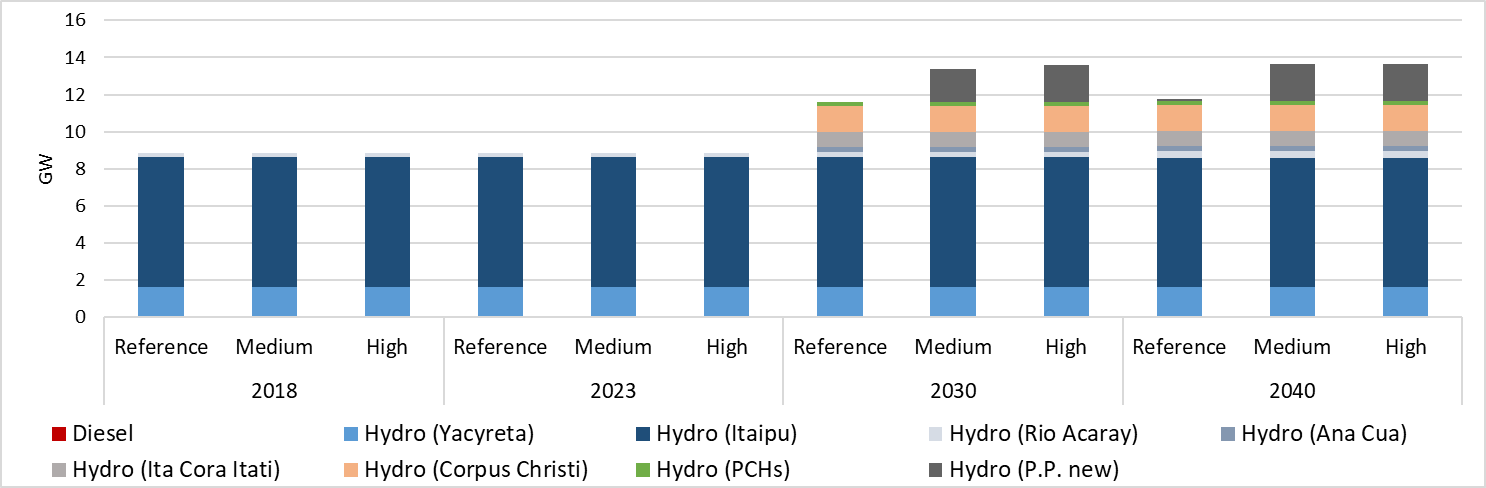


Supplementary Figure 7. Total installed capacity by technology (GW) under the different demand – ISC.3 scenarios.


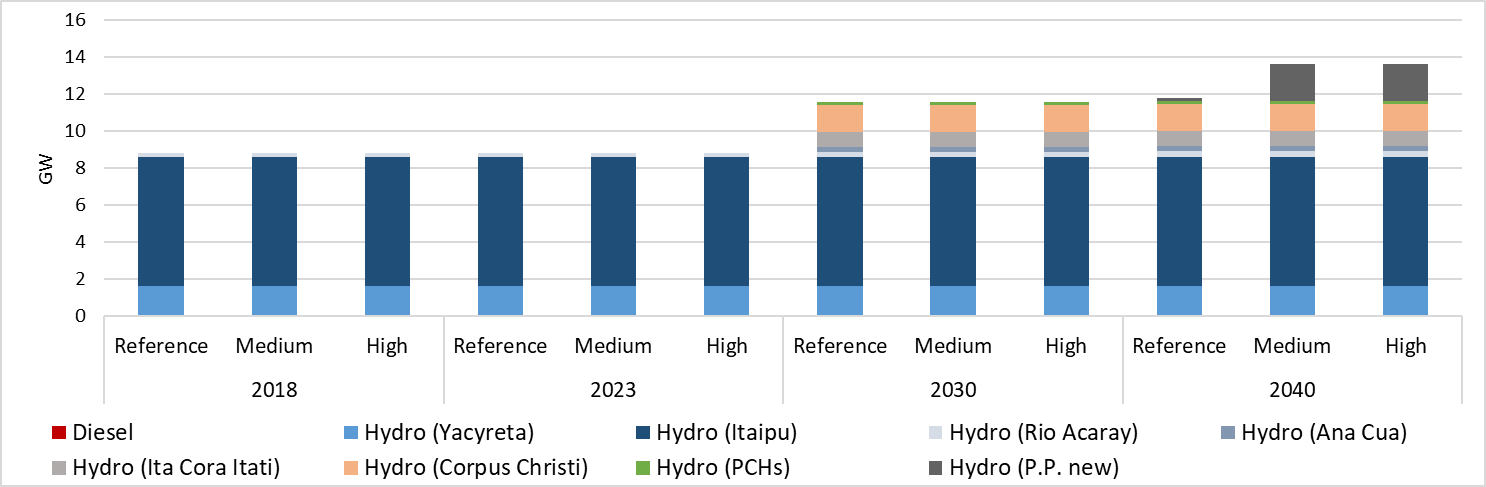


Supplementary Figure 8. Total installed capacity by technology (GW) under the different demand – ISC.4 scenarios.

The electricity generation mix by technology under the different demand combined with electricity export price scenarios is presented in the following figures (Figure 5-8).


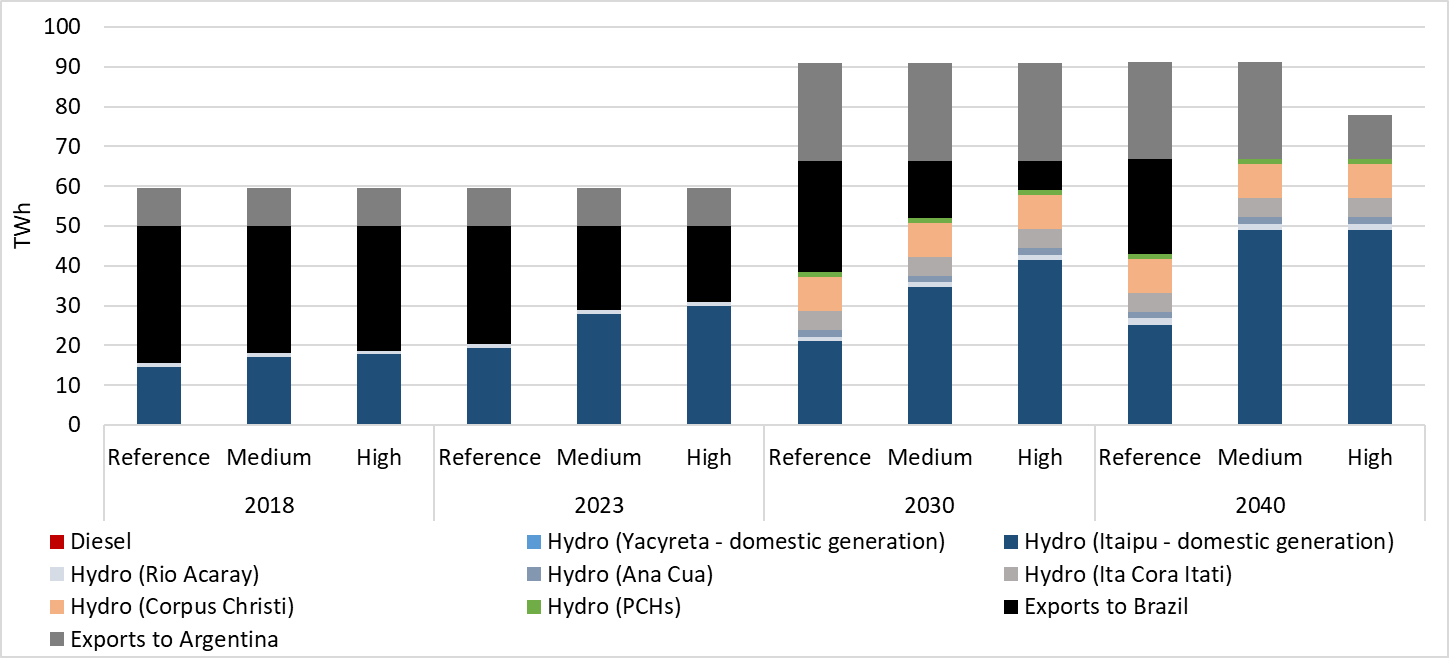


Supplementary Figure 9. Electricity generation mix by technology (TWh) under the different demand – ISC.1 scenarios.


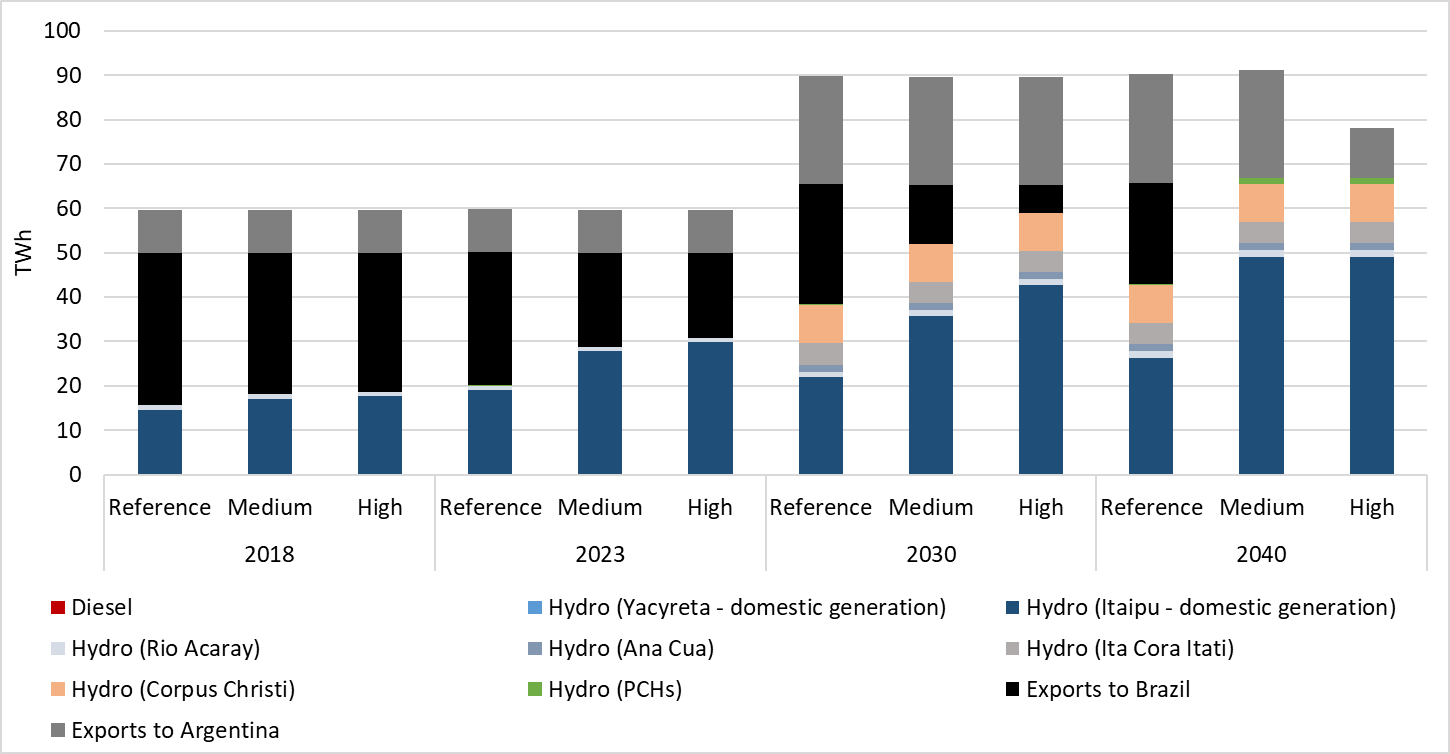


Supplementary Figure 10. Electricity generation mix by technology (TWh) under the different demand – ISC.2 scenarios.


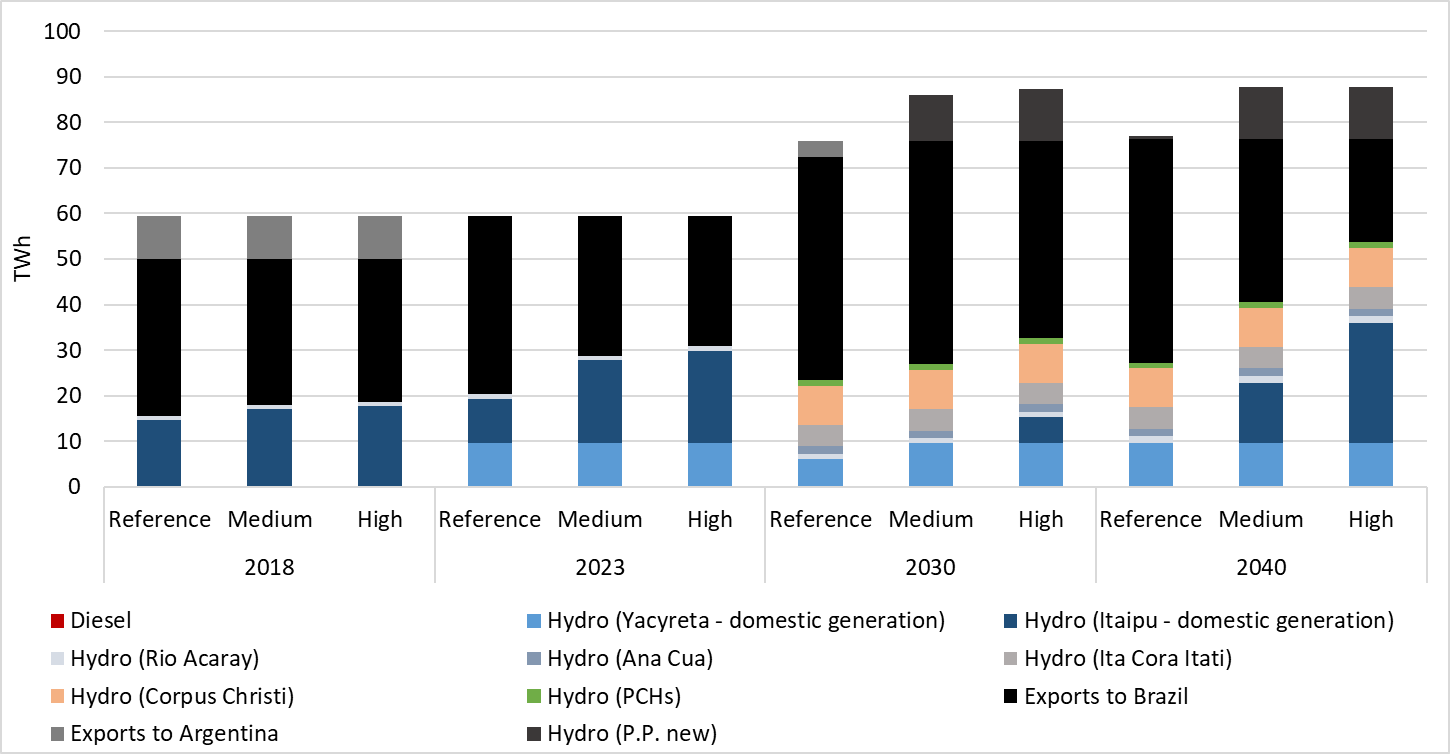


Supplementary Figure 11. Electricity generation mix by technology (TWh) under the different demand – ISC.3 scenarios.


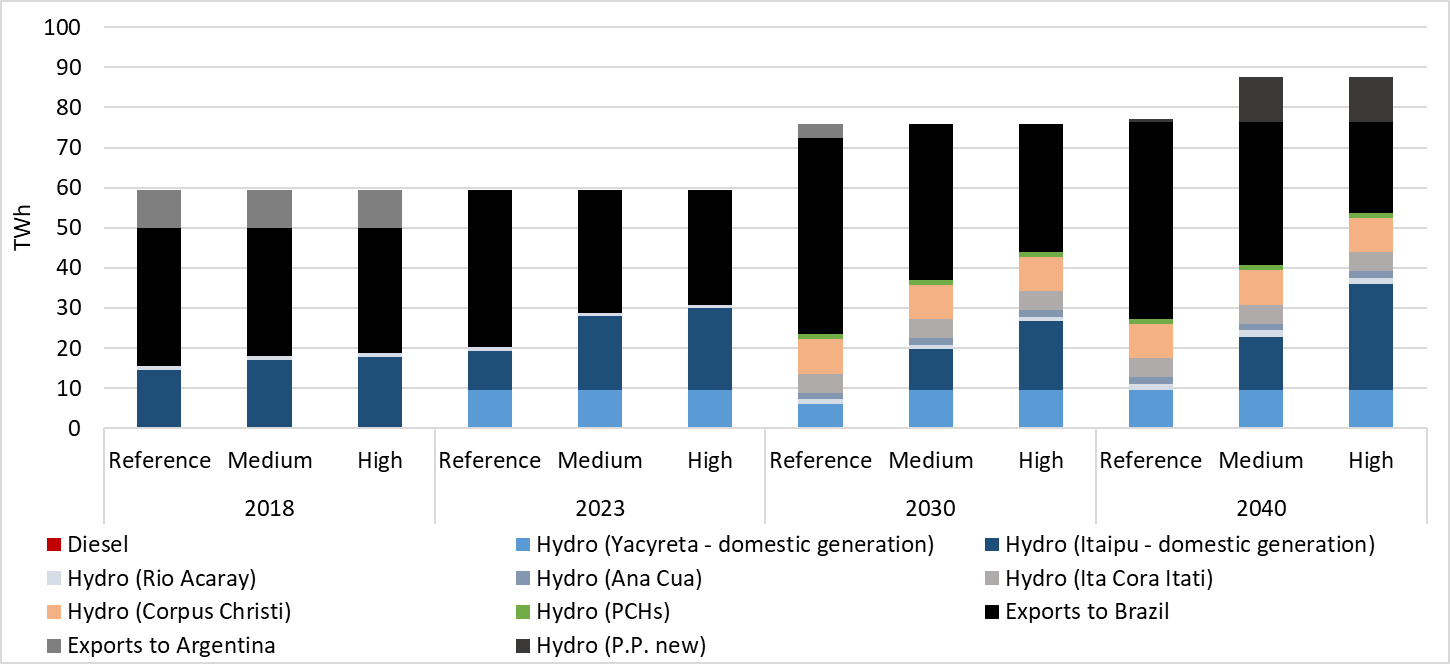


Supplementary Figure 12. Electricity generation mix by technology (TWh) under the different demand – ISC.4 scenarios.

Total profits for Paraguay accordance with the electricity export price of Itaipu for the modeling period 2018-2040.


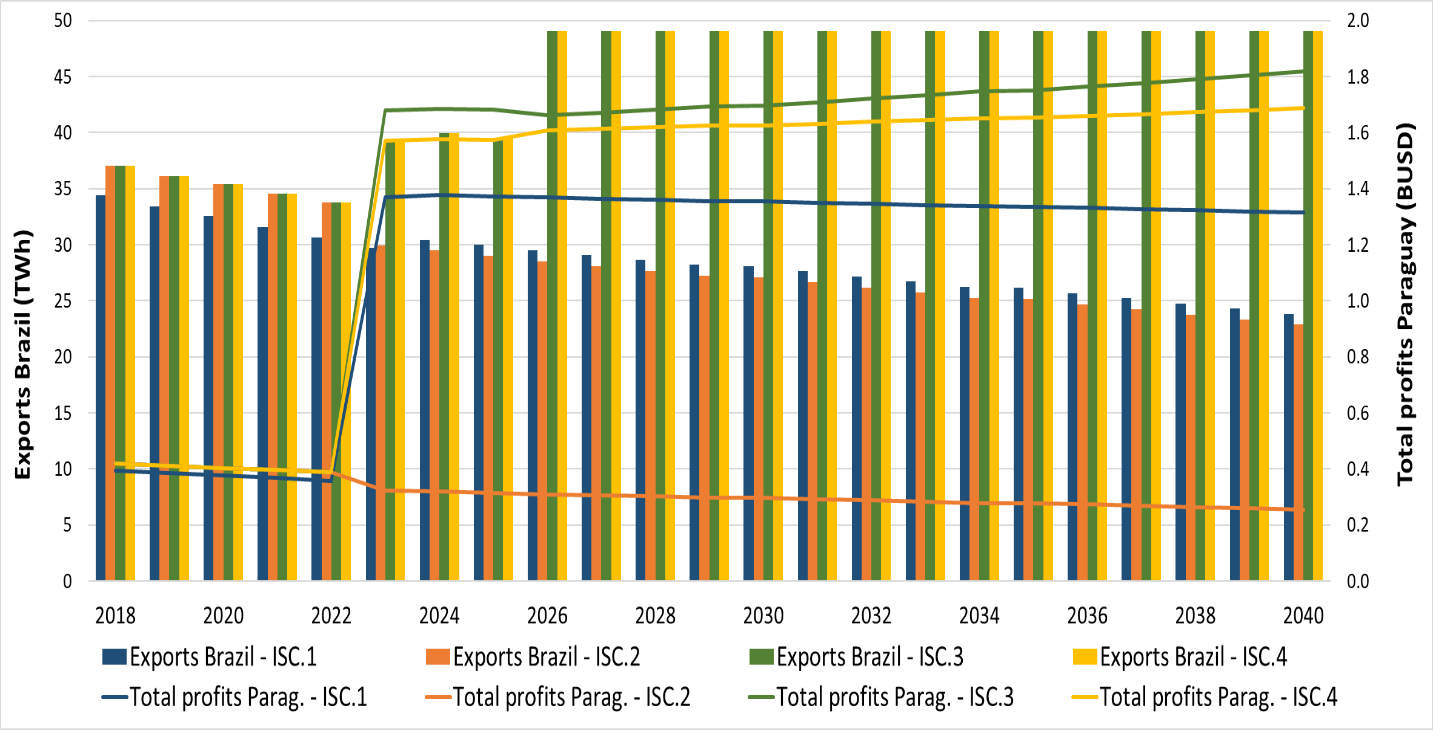


**Supplementary Figure 13.** Reference scenario – Exports to Brazil (TWh) vs Total profits for Paraguay (BUSD) from Itaipu.


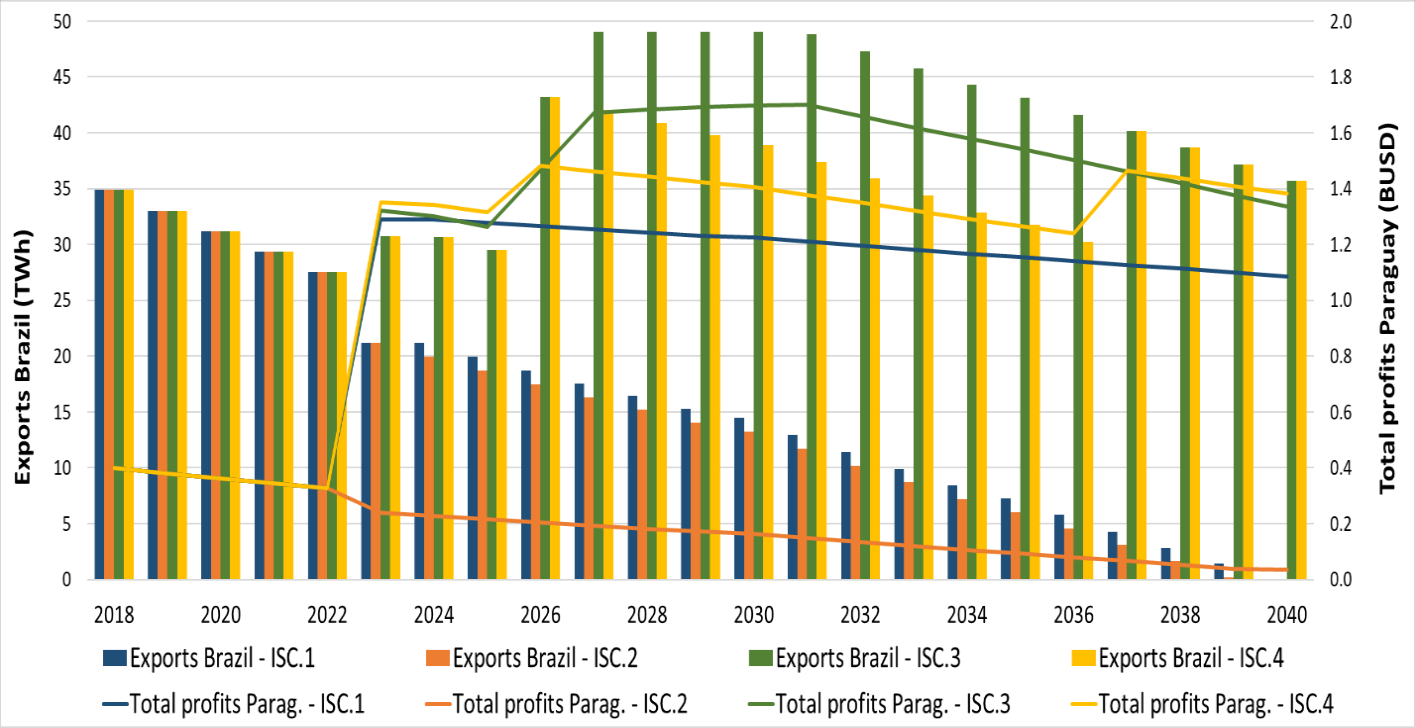


**Supplementary Figure 14**. Medium demand scenario – Exports to Brazil (TWh) vs Total profits for Paraguay (BUSD) from Itaipu.


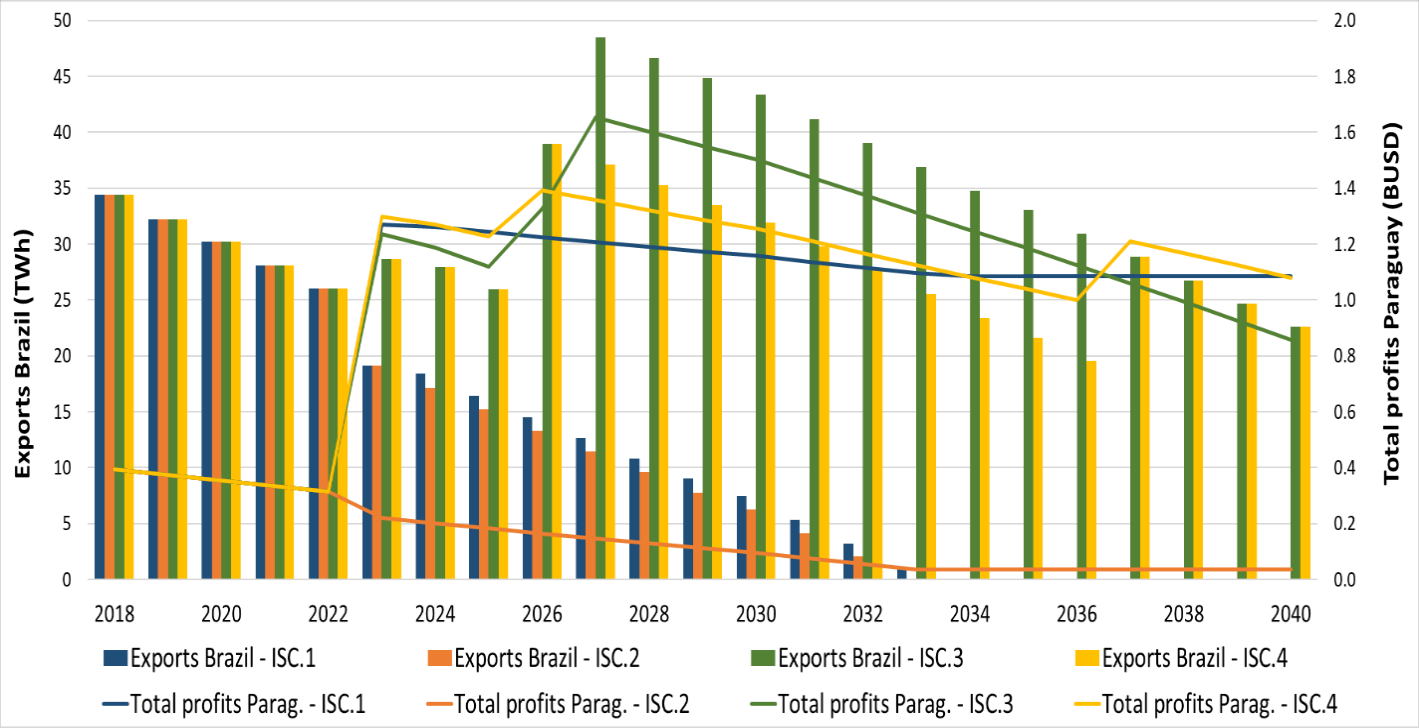


**Supplementary Figure 15.** High demand scenario – Exports to Brazil (TWh) vs Total profits for Paraguay (BUSD) from Itaipu.

References

[1] Administración Nacional De Electricidad, “PLAN MAESTRO DE GENERACIÓN ; PERIODO: 2016 – 2025.” Agosto 2016, Accessed: Sep. 14, 2018. [Online]. Available: http://www.ande.gov.py/documentos/plan_maestro/PM_2016_2025_Gen_Trans_Distrib_Telematica.pdf.

[2] S&P Global Platts, “Latest Oil, Energy & Metals News, Market Data and Analysis | Platts.” 2015, Accessed: May 17, 2018. [Online]. Available: https://www.platts.com/.

[3] IEA, “World Energy Outlook,” 2017, doi: 10.1787/20725302.

[4] Secretaria Tecnica De Planificación Del Desarrollo Económico y Social, “Plan Nacional de Desarrollo Paraguay 2030.” Diciembre 2014, Accessed: Oct. 10, 2018. [Online]. Available: http://www.stp.gov.py/pnd/wp-content/uploads/2014/12/pnd2030.pdf.

[5] COMISIÓN DE INTEGRACIÓN ENERGÉTICA REGIONAL [CIER]., “Síntesis Informativa Energética de los Países de la CIER 2013. Información del sector energético en países de América del Sur, América Central y El Caribe Datos del año 2012.” 2013, Accessed: Nov. 10, 2018. [Online]. Available: https://www.cier.org/es-uy/Lists/EstadisticasLD/Sintesis2013.pdf.

[6] R.A.Landaveri *et al.*, “Elaboración de la prospectiva energética de la República de Paraguay 2013-2040,” Fundación Bariloche, Asunción, Nov. 2015. [Online]. Available: https://www.itaipu.gov.br/sites/default/files/u15/PoliticaE.pdf.
